# Integrated transcriptomic and metabolomic ecotoxicity assessment of earthworms exposed to ZnO and Mn-doped ZnO nanomaterials

**DOI:** 10.3389/ftox.2026.1927574

**Published:** 2026-09-23

**Authors:** Henk J. van Lingen, Paraskevi Zagana, Changlin Ke, Edoardo Saccenti, Marta Baccaro, Maria I. Klapa, Zoi G. Lada, Nico W. van den Brink, Maria Suarez-Diez

**Affiliations:** 1 Laboratory of Systems & Synthetic Biology, Wageningen University & Research, Wageningen, Netherlands; 2 Metabolic Engineering and Systems Biology Laboratory, Institute of Chemical Engineering Sciences, Foundation for Research and Technology-Hellas (FORTH/ICE-HT), Patras, Greece; 3 Division of Toxicology, Wageningen University & Research, Wageningen, Netherlands; 4 Laboratory of Applied Molecular Spectroscopy, Institute of Chemical Engineering Sciences, Foundation for Research and Technology-Hellas (FORTH/ICE-HT), Patras, Greece

**Keywords:** enrichment analysis, gene expression, invertebrate, metabolome, mode of action, multicomponent nanomaterial

## Abstract

**Introduction:**

Zinc oxide (ZnO)-containing nanomaterials may elicit toxic responses in environmental organisms such as earthworms. In this study, we investigate the time-dependent gene expression and metabolomic response in earthworms exposed to ZnO and Mn-doped ZnO (ZnO:Mn) nanomaterials (both at 850 mg/kg soil) and MnCl_2_.

**Methods:**

Earthworms were exposed to ZnO nanomaterial (850 mg/kg soil) for 2, 3, 4, or 7 days, to ZnO:Mn nanomaterial (850 mg/kg soil) for 2, 4, 7, or 14 days, and to MnCl_2_ (45 mg/kg soil) for 14 days. Time-dependent molecular responses were characterized by transcriptomic and metabolomic analyses.

**Results and Discussion:**

Strong differential gene expression was observed after 4 and 2 days of exposure to ZnO and ZnO:Mn, respectively. Moderate differential gene expression was observed after 14 days of exposure to ZnO:Mn and MnCl_2_. Gene ontology (GO) enrichment analysis revealed that differentially expressed genes (DEGs) in earthworms exposed to ZnO were commonly associated with muscles and earthworm movements. Genes upregulated after 2 days of exposure to ZnO:Mn were linked to GO terms associated with microtubule-based structures mediating cellular motility. Downregulated genes were enriched to GO terms among which several were related to energy metabolism. For earthworms exposed to ZnO:Mn and MnCl_2_ for 14 days, few DEGs were involved in GO terms related to diverse biological implications. Aligning with the limited number of GO terms observed, metabolomic disruptions only included increased glutamate and a tendency for increased aspartate in response to MnCl_2_ and ZnO:Mn exposure for 14 days, respectively. Other amino acids and the oxidized-to-reduced glutathione ratio, an indicator of oxidative stress, were not affected. In summary, exposing earthworms to ZnO and ZnO:Mn nanomaterials induced a transient response in differential gene expression related to muscle biology, energy metabolism, and translation, which largely disappeared after 14 days. Combined with minimal metabolomic changes, these findings do not point to substantial long-term ecotoxic effects.

## 1 Introduction

Metal oxide nanomaterials (NMs) such as those containing zinc oxide (ZnO) have become increasingly common resources for a variety of industrial applications (Papadiamantis et al., 2024). Applications of ZnO may include solar cells and semiconductors due to their electrical properties (Özgür et al., 2010), cosmetic products (Lu et al., 2015), UV-blockers in sunscreens (Serpone et al., 2007), and various biomedical applications such as fluorescence bioimaging and anticancer activities (Prashanth et al., 2024). Although ZnO nanomaterials are highly effective, doping them with transition metals such as manganese generates multicomponent nanomaterials (MCNMs) with more distinctive electronic properties. This increases the versatility for applications such as biocatalysis (Aulakh et al., 2022) and fuel cell technology (Hasan et al., 2024). Doping of ZnO with manganese has been suggested as more beneficial than that with other transition metals due to its high thermal stability in the ZnO lattice structure (e.g., Chandekar et al., 2022). Due to the utility of ZnO-containing MCNMs, their toxicity to humans and environmental ecosystems has been screened, but a mechanistic understanding has not yet been obtained (Zhang et al., 2022).

The environmental impact and toxicity of nanomaterials to the soil ecosystem has previously been assessed using earthworms. Exposure of earthworms to Ag_2_S nanoparticles did not affect their burrowing behavior (Baccaro et al., 2019). Nevertheless, exposure of earthworms to bimetallic nanoparticles and combinations of nano- and ionic forms of Au and Ag indicated that dissolution, *i.e*., increased availability of the ionic forms, was a driving factor for metal uptake in earthworms (Baccaro et al., 2022). Co-exposure to both metals in different forms showed different accumulation patterns compared to single-metal exposure, which points to the importance of toxicity testing of chemical mixtures. Furthermore, exposure of earthworms to ZnO NM and ZnCl_2_ at concentrations up to 750 mg Zn kg^−1^ for over 28 days showed that ZnO did not have a clearly harmful effect, whereas ZnCl_2_ was toxic, as indicated by a significant reduction in survival and the subsequent impact on reproductive efficiency (Singh et al., 2024). In line with the latter results, a lower growth constant and lower cellular respiration rate was observed for exposure to ZnCl_2_ than to ZnO after exposure for 98 days (Filipiak and Bednarska, 2021). Nevertheless, ZnO nanoparticles may act as a reproductive toxicant to earthworms at concentrations ≥500 mg kg^−1^, although the exact effect depends on the soil properties (Samarasinghe et al., 2023).

Although various studies have reported commonly applied toxicity tests using earthworms, few studies have attempted to understand the molecular mode of action explaining the observed toxicity. A previously indicated mode of action, though in *Daphnia magna* exposed to ZnO, included altered mitochondrial membrane permeability interfering with the electron transport chain, which may stimulate reactive oxygen species production, detoxify cell mechanisms, and cause dissolution of mitochondria that even lead to cell death (Bacchetta et al., 2016). The mode of action in earthworms exposed to ZnO-containing NM may be assessed through gene expression quantification approaches. Novo et al. (2020) found expression changes of genes encoding for several Zn transporters after 28 days of exposure to ZnO NMs and ZnCl_2_ (*i.e*., Zn in the ionic form) at EC_50_ values in the range from 200 mg–600 mg Zn/kg soil. Furthermore, specific gene expression associated with ZnO NM exposure was related to vesicular transport and specifically to endocytosis. Subsequent analysis indicated enhanced adhesion and peptidase activities linked to a normal physiological function of ionic Zn during epithelial–mesenchymal transition. In addition to the latter relatively long-term exposure study, Gomes et al. (2022) observed a transient gene expression response in potworms after 1–4 days of exposure to ionic Zn from ZnCl_2_ at concentrations of 93 and 145 mg Zn/kg soil. The response was time-dependent with a clear peak in the number of differentially expressed genes (DEGs) on day 3. These DEGs indicated upregulated zinc transporters, oxidative stress, and effects on the nervous system. Moreover, few genes were differentially expressed on multiple days, which indicated a cascade of molecular events occurring in time rather than a sustained response.

The relatively small number of studies that assessed the mode of action in earthworms exposed to ZnO nanomaterials and the absence of studies exploring the gene expression response in earthworms exposed to Mn-doped ZnO (ZnO:Mn) NM warrants further investigation. In addition, metabolomics analyses identify metabolic disruptions and biomarkers of nanotoxicity and offer detailed insights into the downstream physiological changes triggered by nanomaterial exposure (e.g., Yu et al., 2022; Zhu et al., 2020), enabling stronger inference on functional biological effect. Metabolomics complements transcriptomics by assessing if transcriptomic alterations elicit metabolic disturbances and if any ZnO-containing nanomaterial-induced metabolic disturbances occur without any associated transcriptomic alterations. Therefore, integrated transcriptomic and metabolomic findings contribute to a broader understanding of nanomaterial-induced metabolic changes and inform guidelines for safe nanomaterial usage in ecological contexts. This study aims to investigate the dynamics and mode of action of the response of earthworms exposed to ZnO and ZnO:Mn nanomaterials through integrated transcriptomics and metabolomics analysis.

## 2 Materials and methods

### 2.1 Earthworms, soil jar preparation, and experimental design

We present three experiments in which earthworms were exposed to ZnO NM (experiment 1) or to ZnO:Mn MCNM (experiments 2 and 3). Treatments, sampling days, and sample sizes of the design of these experiments are schematically represented in Figure 1. In the first experiment, we exposed earthworms, ordered as *Eisenia fetida* from Dutchworms (Oostwold, Netherlands), to either control or ZnO nanomaterial (Phornano; Korneuburg, Austria)-treated LUFA 2.2 soil (LUFA Speyer, Germany). The supplier indicated that the soil was air-dried and sieved (<2 mm) and had a pH of 5.6 ± 0.29 (mean ± SD), an organic carbon content of 1.82 ± 0.48 w/w%, a measured cation exchange capacity of 9.54 ± 1.36 meq/100 g, and a water holding capacity of 48.9 ± 5.6 g per 100 g. Glass jars were prepared with air-dried soil (150 g per earthworm) spiked with ZnO to reach a nominal concentration of 850 mg/kg, and then additional deionized water (20% w/w, ∼47% water holding capacity) was added. Note that ZnO at 850 mg/kg soil gives rise to the nominal exposure concentration of 683 mg ionic Zn/kg soil. This nominal exposure concentration is slightly greater than the upper bound of 600 mg ionic Zn/kg soil for the reported EC_50_ by Novo et al. (2020), which may amplify toxic responses (Bacchetta et al., 2016) and contribute to the elucidation of the mode action of ZnO NM in organisms. Preparing the control and treatment soils, adding the ZnO nanomaterial and water, and the soil mixing was performed manually. This led to 16 jars with control soil and 16 jars with ZnO-treated soil. After 4 days of equilibration of the soil, jars were refilled with deionized water up to their weight at soil preparation, after which, four clitellated earthworms, *i.e*., sexually matured individuals, with an average weight of 386 ± 39 mg were randomly placed into every jar. Additionally, an amount of 0.5 g of dried horse manure per worm was amended on top of the soil in the jars to feed the earthworms. This horse manure was obtained from an organic farm (Bennekom, Netherlands) with known absence of pharmaceutical use. The soil was periodically moistened using deionized water to maintain the soil moisture at the previously mentioned ∼47% of the water holding capacity throughout the experiments.

**FIGURE 1:**
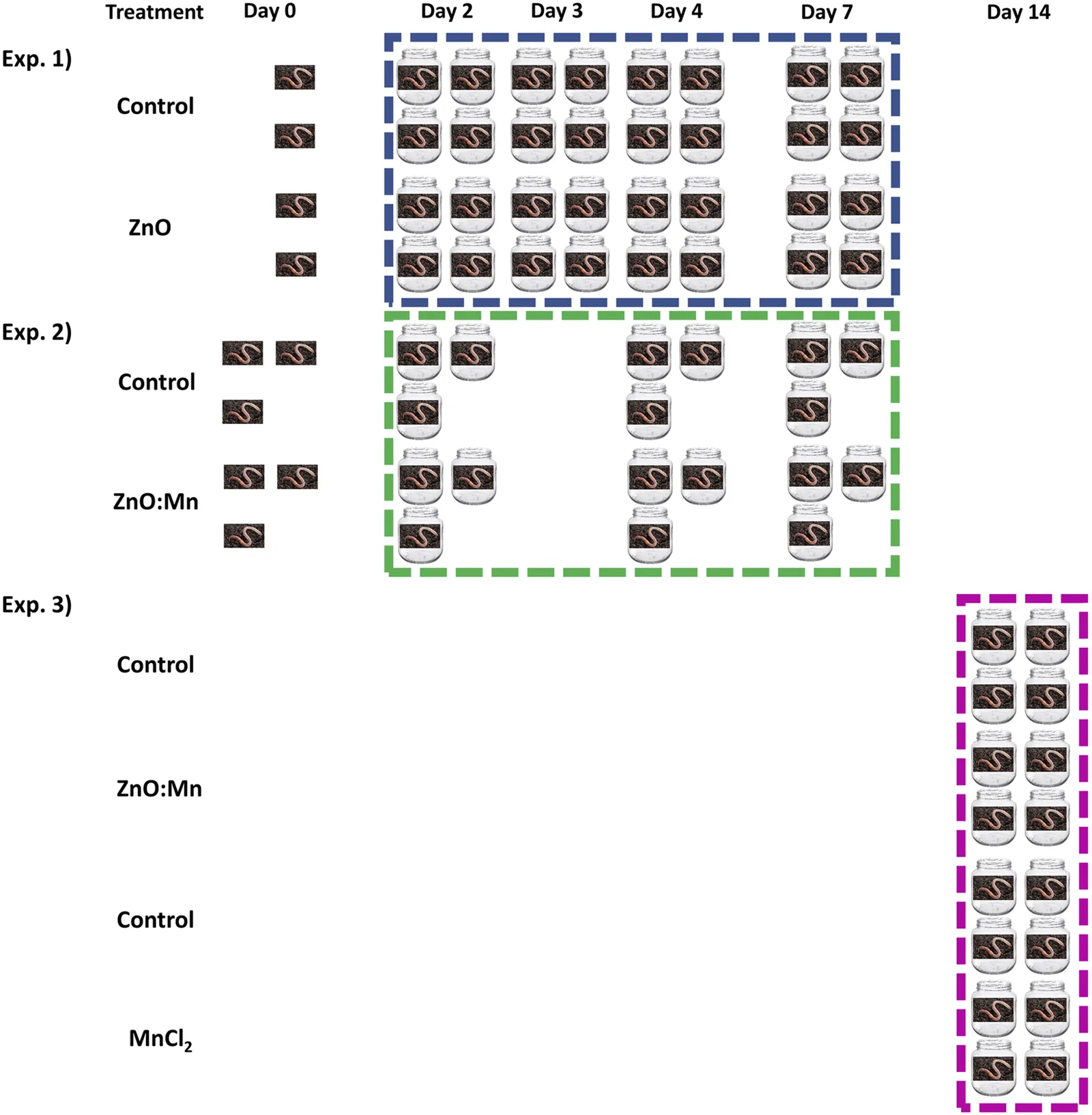
Treatments, sampling days, and sample sizes in earthworm experiments 1, 2, and 3. Dotted squares with jars indicate incubated earthworms. Earthworms shown outside the dotted squares were sampled as reference worms before the start of the incubation on day 0 in experiments 1 and 2.

At the start of the first experiment, four earthworms to be used as baseline references were snap-frozen in liquid nitrogen and stored at −80 ^o^C. Then, throughout the entire experiment, jars containing the prepared soils and earthworms were stored in a climate-controlled cabinet (Weiss Technik, Germany) at 20 ^o^C, 75% air humidity, and a light intensity of 30 µmol/m^2^/s. Lights were switched on for 24 h per day to stimulate the earthworms to stay in the soil and discourage them from escaping from the jars. All 32 jars stayed in the cabinet for 7 days and were positioned in a completely staggered manner with respect to the treatment and time of earthworm sampling using a rectangle of four rows and eight columns. Sampling took place on days 2, 3, 4, and 7 from the start of the exposure. This sampling scheme was inspired by a potworm study (Gomes et al., 2022) in which worms were sampled on days 1, 2, 3, and 4 from the start of the exposure to ZnCl_2_. As limited differential expression was observed on day 1 of that study, we decided to omit sampling on day 1 in our experiments and attempted to sample on days 2, 3, and 4. In addition, we included a sampling on day 7 to assess later responses. Hence, on each of these four sampling days, individual earthworms from four jars with control soil and four jars with ZnO-treated soil were collected, carefully rinsed using deionized water, snap-frozen in liquid nitrogen, and then stored at −80 ^o^C. In addition, approximately 35 g of soil was collected per jar and then oven dried at 70 ^o^C for 24 h. Furthermore, throughout the experiment, jars were refilled with deionized water up to the total weight that every jar with soil and the earthworms had at the start of the exposure. This refill of water was repeated every incubation day apart from the weekend days. After the 7-day exposure period was completed, one collected whole earthworm per jar was taken from the freezer and ground in liquid nitrogen using a mortar and pestle.

The second experiment is also represented in Figure 1 and exposed earthworms, which were also ordered as *Eisenia fetida* from Dutchworms, to either control or ZnO:Mn multicomponent nanomaterial (Phornano; Korneuburg, Austria)-treated LUFA 2.2 soil. Apart from a different nanomaterial and a different number of jars, the experimental conditions were the same as in experiment 1. These other differences were applied to the number of jars and earthworms and the sampling days. In the second experiment, nine jars with control soil and nine jars with ZnO:Mn-treated soil at 850 mg nanomaterial/kg soil were prepared. Note that the 95:5 ratio of ZnO:Mn of the MCNM leads to nominal exposure concentrations of 650 mg ionic Zn/kg soil and 45 mg ionic Mn/kg soil. Thereafter, six clitellated earthworms were snap-frozen in liquid nitrogen and then stored at −80 ^o^C, and two clitellated earthworms with an average weight of 604 ± 63 mg per earthworm were randomly placed into every jar. All 18 jars stayed in the cabinet for 7 days and were positioned in a staggered manner with respect to the treatment and time of earthworm sampling using a rectangle of three rows and six columns. Largely aligning with experiment 1, the samplings took place on days 2, 4, and 7 from the start of the exposure, with the sampling on day 3 omitted due to the limited availability of the ZnO:Mn nanomaterial. Hence, on every sampling day, individual earthworms from three control jars and three ZnO:Mn-treated jars were collected.

To explore longer-term effects, experiment 3 was designed with a 14-day exposure period, representing the endpoint measurement relative to experiments 1 and 2, and it is schematically illustrated in Figure 1. This third experiment of the present study exposed earthworms, which were also ordered as *E. fetida* from LASEBO (Nijkerk, Netherlands), to either control or ZnO:Mn MCNM (Phornano; Korneuburg, Austria)-treated LUFA 2.2 soil or to either control or MnCl_2_-treated LUFA 2.2 soil. The nominal exposure concentration of ZnO:Mn was also set at 850 mg/kg soil, which aimed at quantifiable zinc above the natural background concentration in the soil. Noting the 95:5 ratio of ZnO:Mn of the MCNM, the nominal exposure concentration of ionic Mn from MnCl_2_ was set at 45 mg Mn/kg soil. Four jars each with control soil and ZnO:Mn-treated soil and four jars each with control soil and MnCl_2_-treated soil were prepared. Six clitellated earthworms with an average weight of 584 ± 64 mg were randomly placed into every jar. All 16 jars remained in the cabinet for 14 days, after which all earthworms were sampled at once, depurated from their gut content for 24 h on a petri dish, and then snap-frozen in liquid nitrogen in pairs of two earthworms and stored at −80 ^o^C. Paired earthworms were taken from the freezer and ground in liquid nitrogen using a mortar and pestle. The ground tissue was then divided into two equal amounts, enabling both transcriptomics and metabolomics analysis. Other details regarding the experimental design and sampling were as in the first experiment, in which earthworms were exposed to ZnO.

### 2.2 Nanomaterial characterization

Nanomaterials were characterized using Raman spectroscopy, which is a vibrational spectroscopic technique based on the inelastic scattering of monochromatic light by molecules. It probes molecular vibrational modes, of which the spectrum provides a molecular fingerprint that reflects the chemical composition, molecular structure, and chemical bonding of the sample (*e.g.*, Browne and McGarvey, 2007). Raman spectroscopy identified the most intense peak at 438 cm^−1^ corresponding to the E_2_ mode typical for the hexagonal crystal structure of ZnO nanomaterial samples. Additional less intense peaks were observed at 330 and 580 cm^-1^, and a broad peak was observed at approximately 1,150 cm^-1^. For ZnO:Mn, a less intense peak at 438 cm^-1^ in the Raman spectrum indicates defects and a breakdown in translational symmetry caused by substitutional or interstitial Mn dopant. Additional peaks at 530, 570, and 675 cm^-1^ only observed after Mn-doping of ZnO may reflect a local surface distortion that could influence the reactivity of the nanoparticles (Zhu et al., 2020). Furthermore, X-ray diffraction analysis confirmed the hexagonal structure of ZnO and showed that the doping process reduces the overall crystallinity of the ZnO structure, and broader diffraction peaks pointed to a hexagonal wurtzite (P63 mc) lattice symmetry (Safaei-Ghomi and Ghasemzadeh, 2017). The Mn dopant interferes with the lattice during synthesis, causing more disordered region, reducing the crystal symmetry, and increasing the crystallite size. In addition, scanning electron microscopy image analysis evaluating the crystal morphology indicated that ZnO appears in particles that had a diameter of 50 ± 19 nm, while ZnO:Mn has a surface structure, indicating increased aggregation or particle fusion due to Mn incorporation. The diameter size of ZnO:Mn could not be determined due to its surface morphology. Further details of the experimental characterization of the nanomaterials are provided in the Supplementary Material, along with the Raman and X-ray diffraction spectra and scanning electron microscopy images (Supplementary Figures S1–S3). The present observed lattice strain and defects and the morphological changes induced by Mn-doping enhance the chemical reactivity and bioavailability of the nanoparticles. These properties may influence the extent and mechanisms of biological interaction, including uptake and oxidative stress responses, ultimately inducing the cellular toxicity profile (Persaud et al., 2020). For further details on the ZnO nanomaterial and ZnO:Mn MCNM characterization, which is the Mn-doped analogue of ZnO, we refer to Carreira-Barral et al. (2025) and the Supplementary Material. In addition, the procedure for zinc and manganese content determination in the soil samples is also provided in the Supplementary Material.

### 2.3 RNA-sequencing and downstream data analysis

Ground earthworms from experiments 1 and 2 were sent to BMKGene (Münster, Germany) for RNA extraction and sequencing, while ground samples of two paired earthworms from experiment 3 were sent to GENEWIZ GmbH (Leipzig, Germany). Obtained RNA-seq reads were mapped to both the reference genomes of *E. fetida* (GCA_003999395.1, NCBI; Bhambri et al., 2018) and *E. andrei* (GWHACBE00000000, NGDC/GWH; Shao et al., 2020). Note that the *E. fetida* reference genome is a short-read draft assembly rather than a chromosome-level genome with the median contig length being shorter than a typical gene. This high degree of fragmentation limits its suitability for reliable read mapping and downstream analyses. Therefore, we based our transcriptomic analyses on reads mapped to the higher-quality *E. andrei* reference genome. Samples that mapped >40% to *E. andrei* in experiments 1 and 2 were retained for downstream analysis. This 40% cutoff was set based on five samples in experiment 3 with *E. andrei* mapping rates >40%. For experiment 3, samples with insufficient *E. andrei* reference mapping, a sample-specific *de novo* transcriptome was assembled (Trinity 2.15.1) from all 16 libraries, filtered (TPM ≥3, length ≥250 bp), and functionally annotated using Trinotate 4.0.2 (DIAMOND vs. SwissProt, Pfam) and eggnog-mapper v2.1.12 for GO/KEGG enrichment. This annotation was used exclusively for downstream enrichment analysis of *de novo*-derived DEGs. In addition, BUSCO assessment (v5.7.1, metazoa_odb10, transcriptome mode) of the filtered *de novo* assembly (TPM ≥3, length ≥250 bp; 74,071 genes) yielded 64.4% complete BUSCOs (25.6% single-copy, 38.8% duplicated, 4.0% fragmented, and 31.6% missing). The high duplication rate reflects retained Trinity isoforms and multi-species assembly; CD-HIT clustering did not substantially alter BUSCO metrics. Salmon alignment of reads to the filtered assembly averaged 81.8% (range 78.6%–84.9%). Further technical details regarding RNA extraction and sequencing, raw-reads quality control, read alignment and quantification for all samples, and the reference-free assembly and read counting are provided in the Supplementary Material.

Following RNA sequencing and upstream data analysis, genes with low expression levels across samples were filtered out prior to statistical testing. Low expression was defined as less than 50 reads per gene across samples, except for the five samples mapped to *E. andrei* in experiment 3, for which a cutoff of 25 reads per gene across samples was applied. Normalization of the read counts was performed using the relative log expression method using the DESeq2 package v1.30.0 (Love et al., 2014) in R version 4.4.2. Considering the day × treatment interaction effects for experiments 1 and 2, normalized read counts were fitted using the negative binomial model approach implemented in DESeq2. For experiment 3, only the treatment effects were considered for the negative binomial model as all samplings were taken on the same experimental day. It should be noted that individual earthworms from a unique jar in experiments 1 and 2 and two pooled earthworms from a unique jar in experiment 3 were considered the experimental unit in the statistical analysis. Thereafter, Wald tests were conducted to determine the significance of differential expression for each gene. To correct for multiple comparisons, the Benjamini–Hochberg procedure was applied, leading to adjusted *P*-values. Genes with a log2 fold change greater than 0.58 or less than −0.58, corresponding to a fold change of 1.5 times or 1/1.5 times and an adjusted *P*-value less than 0.05 were considered significantly differentially expressed.

Functional GO enrichment of genes found differentially expressed or selected using regression-based analysis was performed by the hypergeometric function to model the background probability, and the Benjamini–Hochberg procedure was used to correct for multiple testing and generate adjusted *P*-values. Annotated GO terms were downloaded from the KEGG/Gene Ontology database (Kanehisa et al., 2016). GO enrichment analyses were performed using the clusterProfiler package in R (Wu et al., 2021).

### 2.4 Metabolomic sample preparation, data processing, normalization, and filtering

Metabolomic analysis was performed at the Metabolic Engineering and Systems Biology Laboratory (MESBL) of the Institute of Chemical Engineering Sciences, Foundation for Research and Technology-Hellas (FORTH/ICE-HT), Patras, Greece. The homogenized powder samples, obtained through grinding pairs of two earthworms from experiment 3, were shipped from Wageningen University and Research, Netherlands. Technical details regarding metabolite extraction, LC–MS analysis, data acquisition, and metabolite identification are available in the Supplementary Material.

Following chromatogram acquisition, an unexpected cluster of peaks appeared after 18 min–28 min, and an unexpectedly high peak (535.2 m/z at 23.4) appeared in some samples. These unexpected peaks were considered bacterial metabolite background signals due to residual and horse-manure-enriched soil contents and earthworm gut-bacteria. Therefore, these peaks were considered the interference observed in the chromatographic data. Hence, the metabolomic analysis was restricted to only profiling of basic amino acids and molecules eluting outside the interference window.

To address biases arising from sample preparation and the analytical process, total peak areas of metabolites were normalized to the peak area of the norleucine internal standard. Additionally, the peak areas of the metabolites were further normalized to the weight of the ground earthworm samples (105.8 ± 3.9 mg) to ensure comparability. The consistency of the analytical process across all samples was validated by comparing the relative peak area (RPA) profiles of the samples to the RPA values of the QC samples. Metabolite peaks that were inconsistently profiled among the technical replicates were excluded from the analysis. The metabolic profile of each biological sample was quantified as the mean RPA profile of its technical replicates after removing inconsistently determined metabolites. These refined profiles formed the basis for data analysis to derive biologically relevant conclusions.

### 2.5 Statistical analysis of metabolomic data

As each biological sample in experiment 3 represented two earthworms, it was crucial to account for inherent differences initially across the four earthworm samples used in each condition (e.g., MnCl_2_, ZnO:Mn, and their corresponding control treatments). The data were first explored by principal component analysis. In addition, to assess significant differences between the experimental groups, statistical analysis was conducted using general linear modeling, with two pooled earthworms from a unique jar being considered the experimental unit. Residual diagnostics of the fitted models were visually inspected to verify that the model assumptions were satisfied. Thereafter, pairwise comparisons (ANOVA) were performed for the groups (MnCl_2_ vs. its control and ZnO:Mn vs. its control). *P*-values were calculated based on a significance threshold of *P* < 0.05. This approach ensured that variability among individual worms was accounted for, providing a robust assessment of metabolic differences between groups.

## 3 Results and discussion

As schematically presented in Figure 1, three experiments were performed. In experiment 1, earthworms were exposed to ZnO and samples were collected on days 0, 2, 3, 4, and 7 from the start of the exposure. In experiment 2, earthworms were exposed to ZnO:Mn and samples were collected on days 0, 2, 4, and 7. Finally, experiment 3 represents an endpoint measurement in which earthworms were exposed to ZnO:Mn and MnCl_2_ for 14 days until samples were taken. Soil Zn concentrations in experiments 1 was not significantly different from the nominal concentrations set at 683 mg/kg soil (Supplementary Figure S4). In addition, soil Zn concentrations in experiments 2 and 3 along with the soil Mn concentration in experiments 2 and 3 were not significantly different from the nominal concentrations set at 650 and 45 mg/kg for Zn and Mn, respectively (Supplementary Figures S4–S6). In addition, earthworms exposed to ZnO:Mn for 14 days in experiment 3 had Zn concentrations that exceeded twice the baseline values, whereas Mn concentrations in earthworms exposed to MnCl_2_ for 14 days showed a more moderate increase (Van Lingen et al., 2025).

The present study is unique because we combine 3 experiments that, individually or taken together, track the transient transcriptional response in earthworms exposed to ZnO nanomaterial and ZnO:Mn multicomponent nanomaterial along multiple days for a week. Both by the treatment and sampling days, these two experiments complement a previous study that quantified gene expression in potworms exposed to ionic zinc in the form ZnCl_2_ on days 1, 2, 3, and 4 from the start of the exposure (Gomes et al., 2022). Furthermore, in addition to studies in which earthworms were exposed to ionic zinc and ZnO nanomaterials for 28 days (Novo et al., 2015; Novo et al., 2020), the endpoint measurements after 14 days in our third experiment are complementary by treatment using the ZnO:Mn multicomponent nanomaterial and ionic Mn^2+^ in the form of MnCl_2_. The latter MnCl_2_ and ZnO treatments may be considered positive controls of the ZnO:Mn multicomponent treatment. In addition to gene expression analysis on samples from experiments 1, 2, and 3, the current study performed metabolomic profiling on samples of earthworms exposed to MnCl_2_ and ZnO:Mn for 14 days in experiment 3. This endpoint metabolic profiling further assessed the molecular impacts in addition to endpoint gene-expression analysis.

Several limitations should be considered when interpreting the findings of this study. Although the temporal design enabled identification of transient molecular responses at days 2, 3, 4, 7, and 14 from the start of the exposure, molecular changes observed at specific time-points may not fully capture long-term biological effects. In addition, laboratory exposure conditions and nominal concentrations cannot fully reproduce the complexity of environmentally relevant exposure scenarios, where nanomaterial transformation, bioavailability, and interactions with soil components may differ substantially. A word of caution applies to the use of continuous illumination, which could reduce above-ground activities and may alter the basal physiology of earthworms (e.g., Cai et al., 2025), and to the different earthworm weights used in the three experiments. However, both control and treatment earthworms experienced continuous illumination in all three experiments, and multiple comparisons were only made within study. Therefore, any weight or light-induced changes are unlikely to confound treatment-specific transcriptomic responses. Furthermore, commonly used toxicological response variables such as survival, growth, reproduction, and behavioral responses were not assessed, and conclusions drawn from the present study exclusively apply to molecular responses. Nevertheless, the integrated use of temporal transcriptomics and endpoint metabolomics facilitates a deeper understanding of nanomaterial-induced metabolic changes in earthworms. This framework provides a valuable approach for mechanistic nanotoxicology assessment across organisms and supports the generation of relevant omics datasets for future risk assessment.

### 3.1 Earthworm transcripts mapped to reference genomes and *de novo* assembly

The morphology of *E. fetida* earthworms is typically characterized as striped, whereas *E. andrei* are described by uniform dorsal pigmentation (Sheppard, 1988). However, analyses based on coelomic fluid fluorescence (Plytycz et al., 2024) and DNA barcoding (Römbke et al., 2016) have demonstrated that morphological appearance does not exhaustively determine the species. On morphology, specimens of molecularly determined *E. fetida* clusters were always identified as *E. fetida*, whereas specimens of molecularly determined *E. andrei* clusters were also identified as *E. fetida*. In addition, *E. andrei* or *E. fetida* specimens produce hybrid offspring (Plytycz et al., 2020), containing genetic information from both *Eisenia* species. However, hybrid genetic information may decrease mapping rates to *E. fetida* and *E. andrei* reference genomes, which could complicate transcriptomic analyses and require *de novo* transcriptomic approaches as applied to experiment 3 in the present study. Moreover, although exposure of hybrid earthworms to Cd impaired cocoon production, earthworm viability was not affected (Jaskulak et al., 2021). The latter may indicate that hybrids may still be suitable for use in toxicological exposure studies depending on the outcome measure used for evaluation.

Most samples in experiments 1 and 2 had mapping rates >40% to *E. andrei* and were retained for downstream analysis (Figures 2A,B). This 40% cutoff naturally appeared from the five samples in experiment 3 that stood out with *E. andrei* mapping rates >40% (Figure 2C). Mapping rates of reads per sample for the three experiments are available in File S1. For experiment 1, the 40% cutoff led to only 1 control sample being retained for the samples obtained on day 4 from the start of the exposure. Therefore, all control samples obtained for day 4 were excluded. For experiment 3, low mapping rates to both *E. andrei* and *E. fetida* for 11 out of the 16 samples (Figure 2C) indicated that the collection of earthworms included at least three species. This mixed-species population is a limitation of the present study, which complicates attribution of transcriptomic responses to a single species. In the past, genotyping was performed using cytochrome amplicon sequencing (e.g., Huang et al., 2007), which could have identified the earthworm species used in experiment 3. Here, we present a *de novo* assembly, which is a straightforward alternative that allows capturing the genetic potential of the (possibly mixed) population. Mapping the transcripts of each earthworm sample to its respective reference genome would generally produce the most robust mapping, but such an approach would have led to transcript samples mapped to multiple genomes that could not be directly compared to each other. The present *de novo* assembly that we performed enables the direct comparisons of transcript samples across species. In addition, because mixed cultures are common in laboratory and applied contexts, our findings are likely representative of conditions frequently encountered in practice. Future studies using verified single-species cultures and chromosome-level reference genomes may further refine the patterns observed here.

**FIGURE 2:**
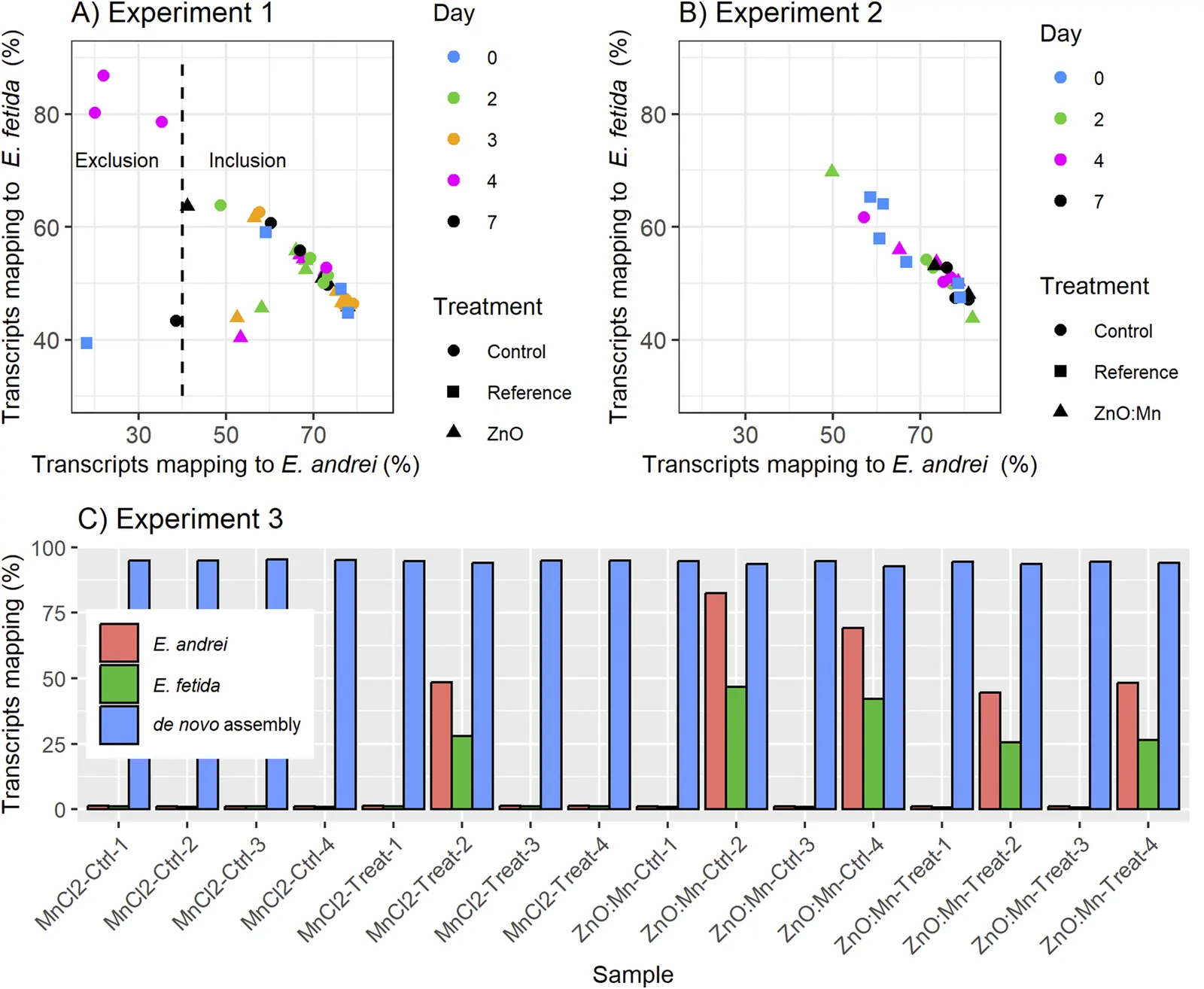
RNA transcript mapping rate per sample to **(A)** *E. andrei* and *E. fetida* in experiment 1, where the dashed line indicates the inclusion and exclusion regions, **(B)** *E. andrei* and *E. fetida* in experiment 2, and **(C)** *E. andrei*, *E. fetida*, and a *de novo* constructed assembly in experiment 3.

The performed *de novo* assembly led to a mapping rate of 94.5% (range 92.8%–95.5%) to the constructed reference transcriptome. Correlation heatmaps did not indicate any patterns or suspicion for the retained samples in experiments 1 and 2 (Supplementary Figures S7-S8). However, when evaluating the *de novo* assembled reads for experiment 3, the five samples, which mapped the highest to the two *Eisenia* species, correlated rather well to each other, but they showed low correlations relative to the remaining 11 samples, which correlated highly to each other (Supplementary Figure S9). Hence, a second read count matrix was generated for these five samples based on the *E. andrei* reference genome instead of the *de novo* assembly. With reference to Figure 2C and Supplementary Figure S9, the 11 *de novo* analyzed samples included four control MnCl_2_ samples vs. three MnCl_2_ treatment samples and two ZnO:Mn control vs. two ZnO:Mn treatment samples. The five samples mapped to the *E. Andrei* reference genome include the remaining two ZnO:Mn control samples vs. two ZnO:Mn treatment samples and one MnCl_2_ treatment sample that was no longer used for downstream analysis.

### 3.2 Differential gene expression analysis

DEGs were defined by adjusted *P*-values less than 0.05 and a threshold fold change greater or less than 1.5 and 1/1.5 (corresponding to 0.585 in log_2_) for identifying biologically meaningful DEGs (Schurch et al., 2016). The number of DEGs in earthworms after 2, 3, and 7 days of exposure to ZnO was very limited. Out of a total of 28,659 genes, the highest number of DEGs observed on day 7 was only 34, of which eight were downregulated and 26 were upregulated (Figures 3A–C; all DEGs are provided in File S2). In line with these DEGs, a regression-based and analysis indicated a cluster of only 13 genes and a cluster of only three genes that had increased expression on days 3 and 7 for ZnO-treated earthworms, respectively (Supplementary Figure S11). Given that no control samples taken on day 4 were retained, this analysis could not be performed on samples from earthworms exposed to ZnO for 4 days. Instead, samples taken at the different days after exposure were compared to the baseline reference samples taken on day 0. This comparison showed 60, 66, 1,079, and 52 DEGs on days 2, 3, 4, and 7, respectively, indicating a sharp peak in gene expression on day 4, of which the majority was downregulated (Figure 4). Furthermore, in line with the latter observation, comparing the ZnO-treated samples taken on day 4 to ZnO-treated samples taken on days 2, 3, and 7 also indicated 697, 385, and 366 DEGs, respectively (Supplementary Figure S10), with the large majority being downregulated. These genes, which were even indicated to be differentially expressed relative to earthworms treated with ZnO taken on days 2, 3, and 7, further indicate a strong temporary gene expression response after 4 days of exposure to ZnO. Although some caution needs to be taken due to the absence of control samples for day 4, a temporary substantial dysregulation of gene expression declining later in time aligns with peak transcriptional responses after 3 days in enchytraeids (potworms) exposed to ZnCl_2_ (Gomes et al., 2022) and earthworms exposed to silver nanomaterials (Tsyusko et al., 2012), which was observed previously.

**FIGURE 3:**
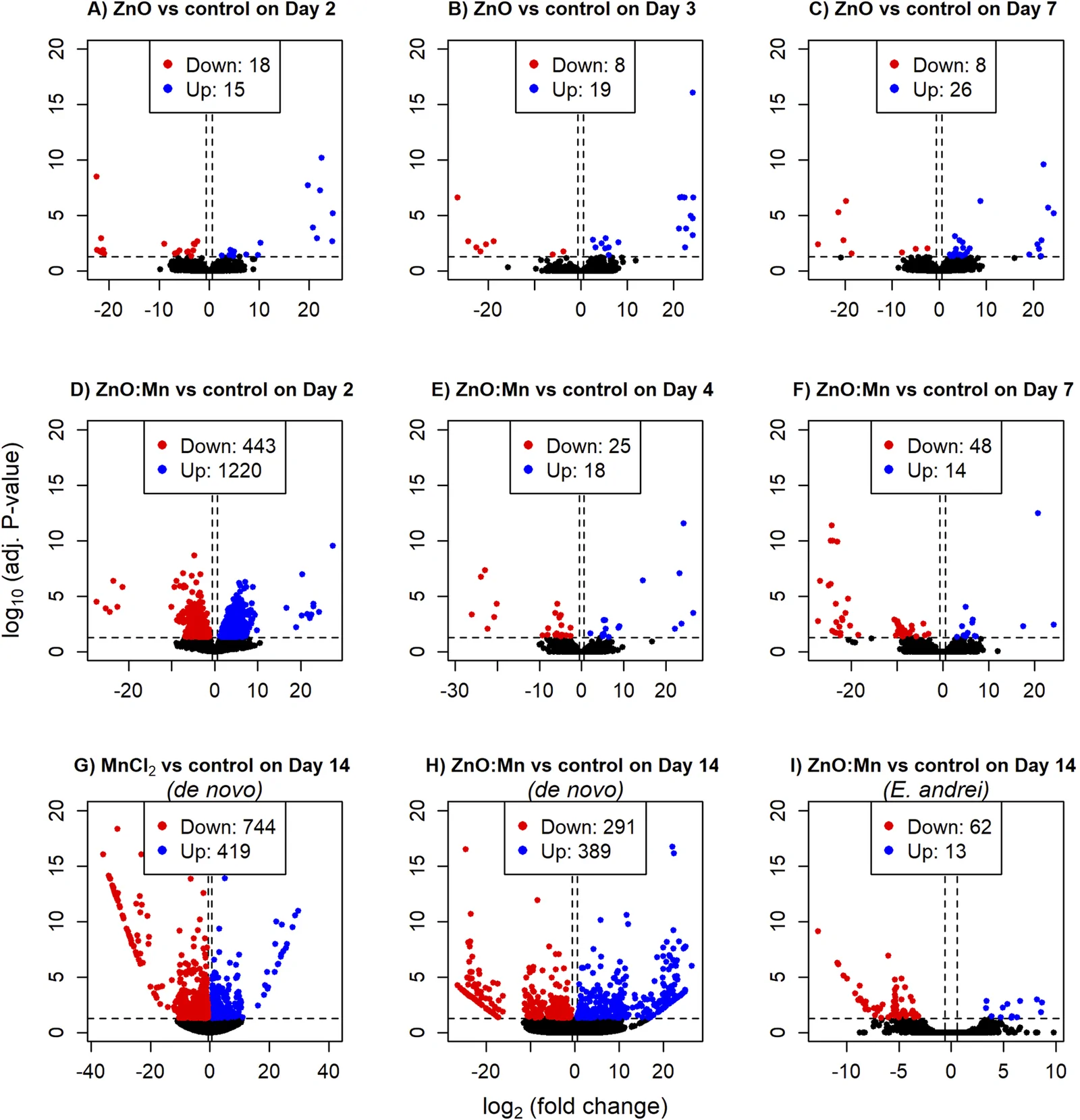
Volcano plots that represent differential gene expression of transcripts mapped to the *E. andrei* reference genome obtained from earthworms exposed to control vs. ZnO-treated soil for 2, 3, and 7 days in experiment 1 **(A–C)**, from earthworms exposed to control vs. ZnO:Mn-treated soil for 2, 4, and 7 days in experiment 2 **(D–F)**, of transcripts assembled *de novo* from earthworms exposed to control vs. MnCl_2_-treated soil and control vs. ZnO:Mn-treated soil for 14 days in experiment 3 (*de novo*) **(G, H)**, and of transcripts mapped to the *E. andrei* reference genome obtained from earthworms exposed to control vs. ZnO:Mn-treated soil for 14 days in experiment 3 (*E. andrei*) **(I)**.

**FIGURE 4:**
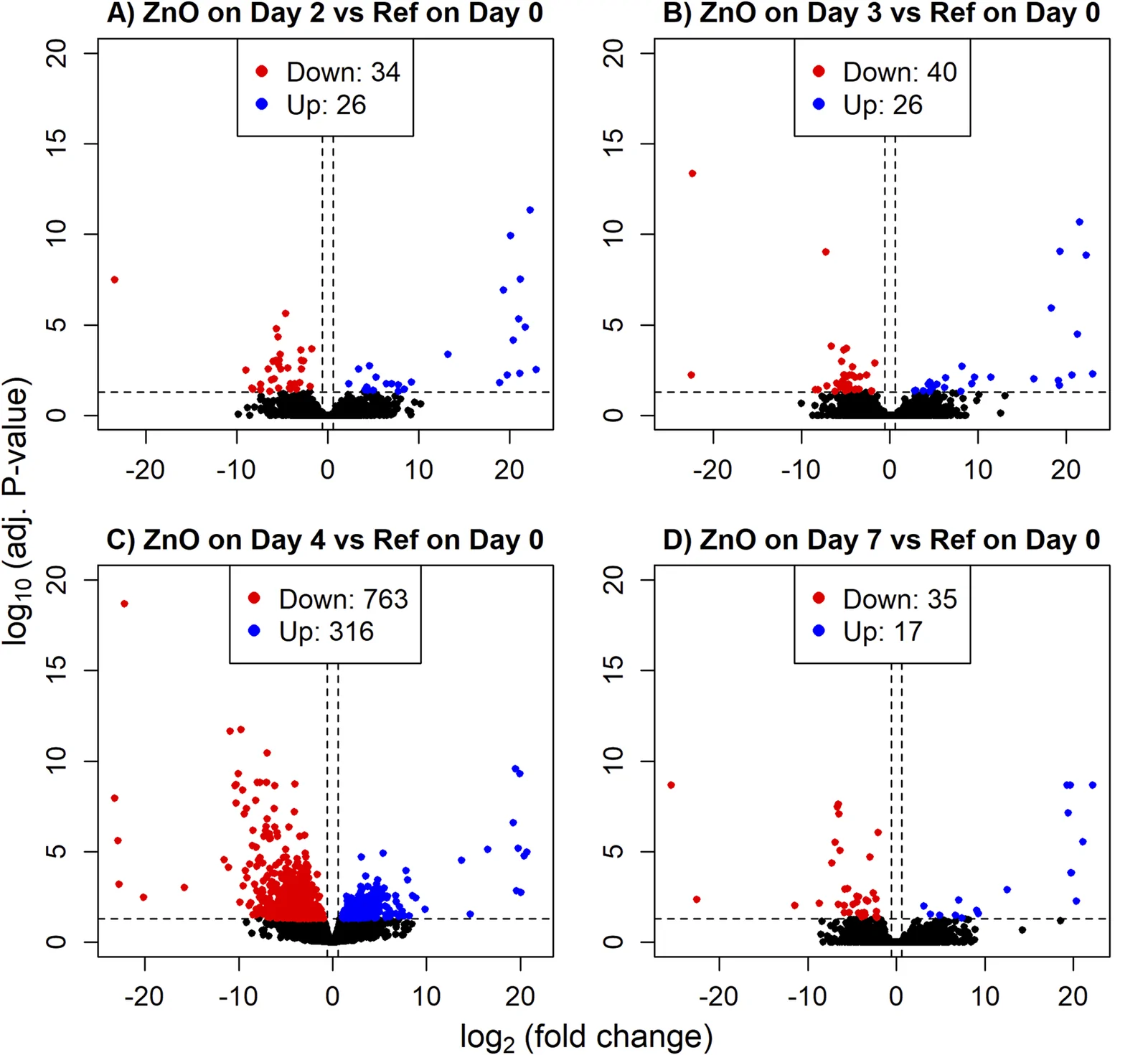
Volcano plots that represent differential gene expression of transcripts mapped to the *E. andrei* reference genome for earthworms exposed to ZnO-treated soil for 2, 3, 4, and 7 days **(A–D)** vs. baseline reference earthworms collected at the start of the exposure on day 0 in experiment 1.

Earthworms exposed to ZnO:Mn had 1,663 DEGs out of 28,509 after 2 days, of which 443 were downregulated and 1,220 were upregulated (Figure 3D). Regression-based analysis indicated a cluster of 167 transcriptionally disturbed genes on day 2 from the start of exposure. These disturbances were largely absent on day 7 (Supplementary Figure S11). This transcriptional response did not persist as only 43 and 62 DEGs were observed on days 4 and 7 from the start of exposure, respectively (Figures 3E,F). The temporary increase in DEGs after 4 days of exposure to ZnO and 2 days of exposure to ZnO:Mn may align with other previous temporary increases in DEGs such as that observed after 3 days of exposure to ionic zinc in potworms (Gomes et al., 2022) and exposure to silver nanomaterials in earthworms (Tsyusko et al., 2012). Exposing earthworms, which are difficult to identify phenotypically, to MnCl_2_ or ZnO:Mn for 14 days indicated 1,163 (744 downregulated and 419 upregulated) and 680 (291 downregulated and 389 upregulated) out of the 46,238 genes annotated in the *de novo* assembled transcriptome, respectively (Figures 3G,H). These DEGs were identified using the 11 samples in experiment 3 showing low mapping to the two *Eisenia* species, which included four control MnCl_2_ samples vs. three MnCl_2_ treatment samples (DEGs in Figure 3G) and two ZnO:Mn control samples vs. two ZnO:Mn treatment samples (DEGs in Figure 3H), and were assembled *de novo*. Although Trinity *de novo* assembly may be a powerful approach, which was demonstrated previously (e.g., on earthworms exposed to copper; Yu et al., 2022), these DEGs may be incomparable to DEGs of *E. andrei* in experiments 1 and 2. However, reads for the two control vs. two ZnO:Mn-treated samples of experiment 3 mapped to the *E. andrei* reference genome are comparable to the gene expression profiles in experiments 1 and 2 based on the reference genome. These samples indicated only 75 DEGs out of 25,984 genes (62 downregulated and 13 upregulated; Figure 3I). The differential expression observed in earthworms exposed to ZnO:Mn for 14 days may need to be interpreted cautiously as the results in both Figures 3H,I are based on only two vs. two samples.

### 3.3 Gene ontology enrichment

DEGs for the various treatments and sampling days underwent enrichment of terms from the Gene Ontology (GO) database (The Gene Ontology Consortium, 2019). For earthworms exposed to ZnO, no significant GO terms were obtained among the DEGs at days 2, 3, and 7 from the start of the exposure. For genes downregulated after 4 days of exposure to ZnO, 93 GO terms were found enriched (adjusted *P*-values <0.05), of which the top 15 on adjusted *P*-value are shown in Figure 5. GO terms comprised various actin (striated) muscle cells, contractile fiber, myofibril, sarcomere, and supramolecular cellular components. Additional details of the enriched GO terms annotated to the DEGs after 4 days of exposure to ZnO are provided in File S3.

**FIGURE 5:**
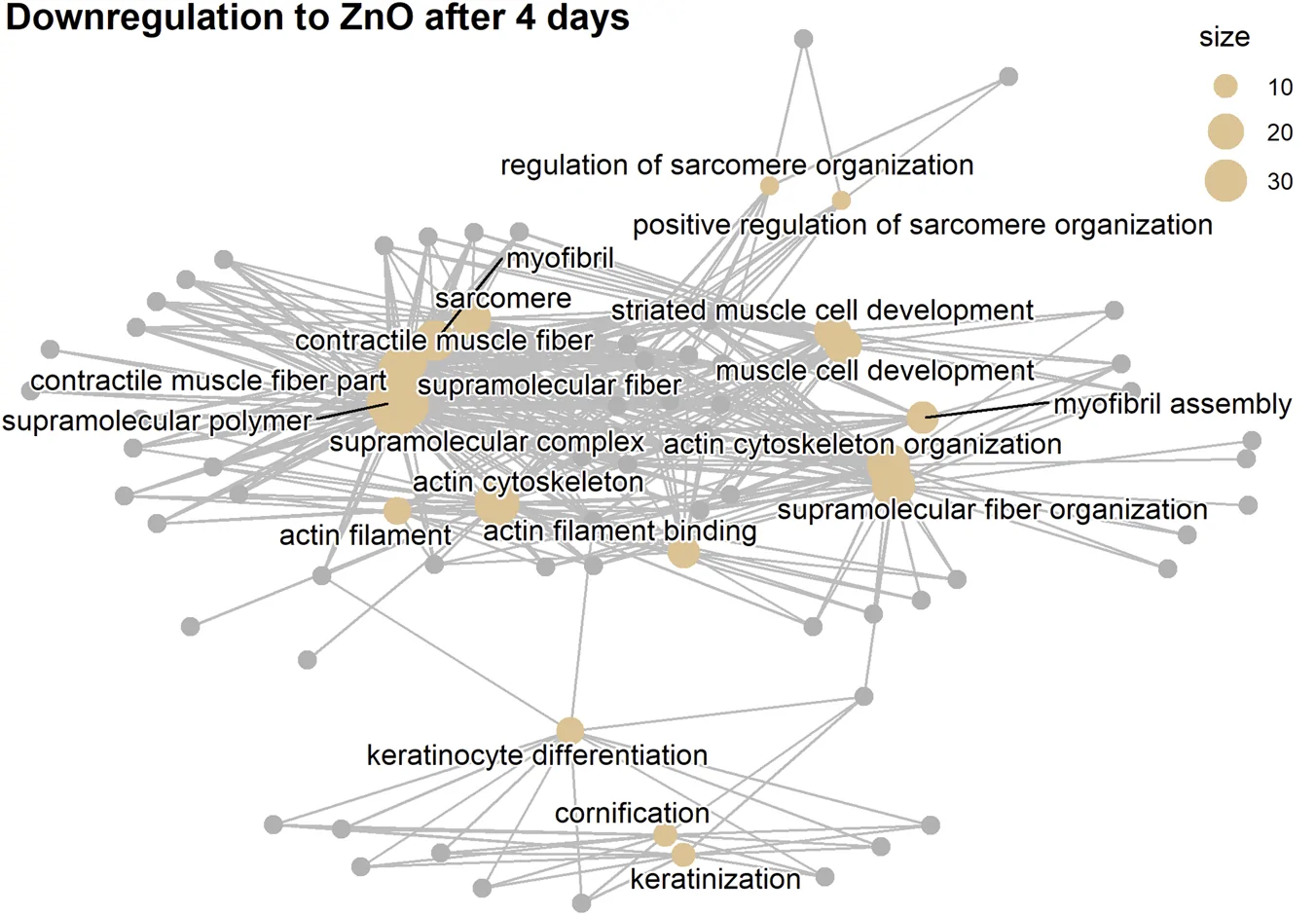
Networks of enriched genes (gray circles) and the top-15 Gene Ontology (GO) terms (beige circles; size represents the number of genes associated to each enriched GO term) for downregulated genes in earthworms exposed to ZnO for 4 days in experiment 1. Gray edges connect genes to GO terms.

When considering DEGs after 2 days of exposure of earthworms to ZnO:Mn, 27 GO terms were enriched among the upregulated genes and five were enriched among the downregulated genes, of which the top 15 are provided (Figures 6A,B). These 27 and five GO terms were associated with up to 41 and 10 genes, respectively. GO terms enriched among the upregulated genes comprised terms associated to various cilium, microtubule-based, cell projection, axoneme, and sperm flagellum from the cellular component (CC) ontology. In contrast, GO terms enriched among the downregulated genes on day 2 comprised terms from the biological process (BP) and molecular function ontologies, in addition to terms from the CC ontology. These GO terms were related to ribosomes, mitochondria, translation, peptide processes, respiration, and oxidative phosphorylation. Novo et al. (2020) also analyzed transcriptional responses of *E. fetida* earthworms exposed to ZnO NM, but they reported enrichment of GO terms that included vesicular/membrane transport, compound binding, adhesion, extracellular processes, and peptidases, which largely do not align with the present GO terms obtained after 4 and 7 days of exposure to ZnO or 2 days of exposure to ZnO:Mn. Moreover, upregulated genes after 4 days and downregulated genes after 7 days of exposure to ZnO:Mn showed enrichment of 80 and 96 GO terms, respectively. However, these were associated to no more than two genes per GO term, and their relevance cannot be ascertained. Finally, upregulated and downregulated genes after 14 days of exposure to ZnO:Mn were enriched in 24 and 50 GO-terms, respectively (File S3). Upregulated and downregulated genes after 14 days of exposure to MnCl_2_ were enriched in 65 and five GO terms, respectively (File S3). Enriched GO terms among the DEGs after 14 days of exposure were associated with no more than four genes, which indicates that these are not systemic effects. These results at the endpoint observation after 14 days of exposure to ZnO and ZnO:Mn nanomaterials and MnCl_2_ may indicate that the applied materials are not overtly toxic to earthworms, which is in contrast to Ag ions or Ag nanomaterials, for which toxicity was observed after 28 days of exposure (Novo et al., 2015).

**FIGURE 6:**
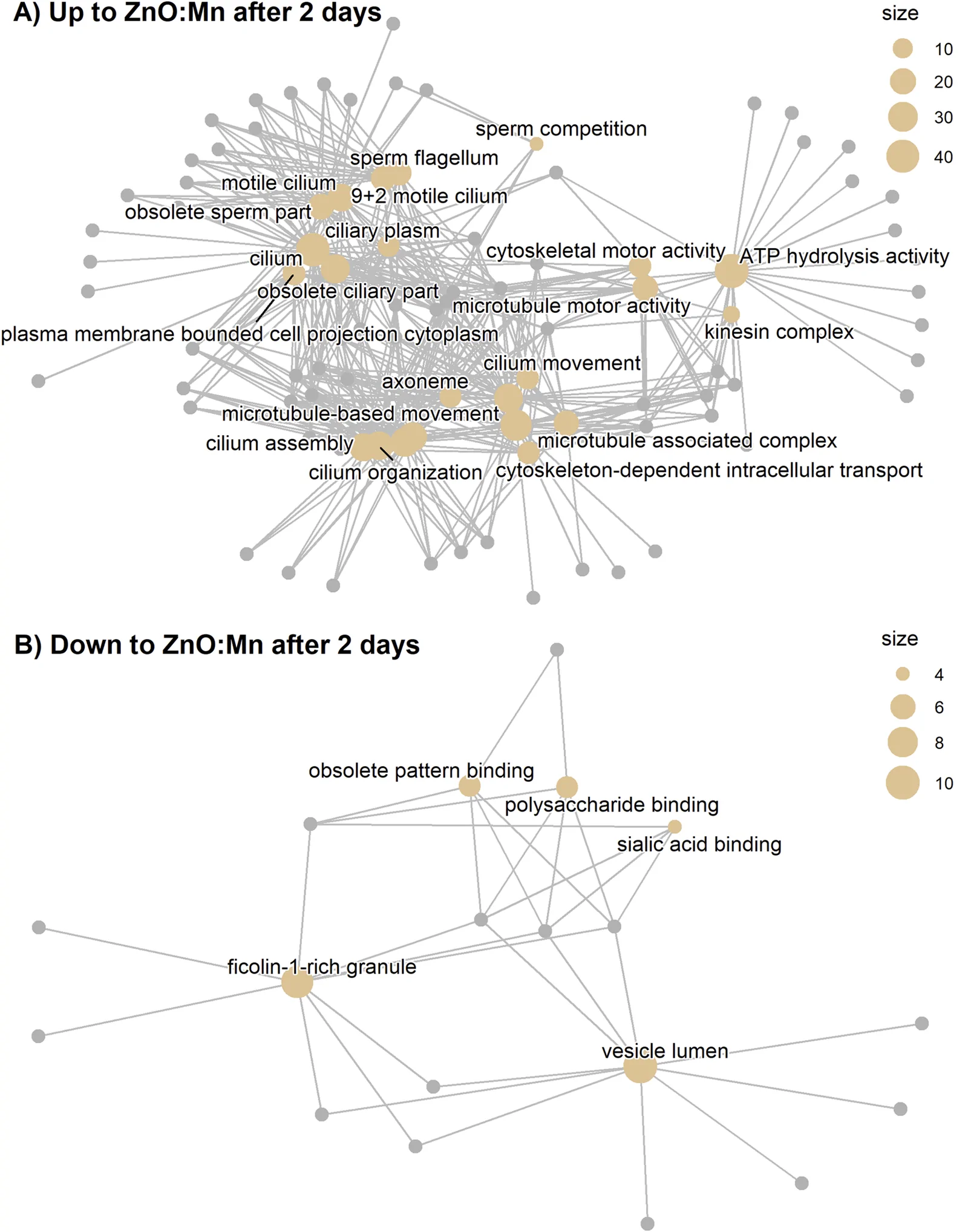
Networks of enriched genes (blue circles) and top-15 Gene Ontology (GO) terms (black circles; size represents the number of genes associated to each enriched GO term) for **(A)** downregulated genes in earthworms exposed to ZnO:Mn for 2 days and **(B)** upregulated genes in earthworms exposed to ZnO:Mn for 2 days in experiment 2. Gray edges connect genes to GO terms.

### 3.4 Metabolites identified after 14 days of exposure to ZnO:Mn and MnCl_2_

The metabolomics analysis, which was only performed for experiment 3, focused on identifying specific biomarkers of nanotoxicity, leveraging high-throughput LC–MS techniques to assess changes in metabolic profiles. These findings may contribute to a broader understanding of nanomaterial-induced metabolic changes and inform guidelines for safe nanomaterial usage in ecological contexts. A PCA of the metabolomic profiles, *i.e.*, relative peak areas, across the four treatment groups (MnCl_2_ control, MnCl_2_ treatment, ZnO:Mn control, and ZnO:Mn treatment) is shown in Figure 7A. The first two principal components explain a substantial proportion of the total variance, together accounting for ∼74% of the variation in the data. Aligning with limited number of GO terms observed in the transcriptomics analysis in experiment 3, there is no clear separation among the treatment groups, indicating that either Zn and Mn exposure has limited influence on the overall metabolic profile or that any effect, if present, is confined to a subset of the measured metabolites or pertains to metabolites that were not measured. Relative peak areas (RPAs) of oxidized glutathione (GSSG), reduced glutathione (GSH), and amino acids in earthworms exposed to MnCl_2_ and ZnO:Mn and the corresponding control treatments for 14 days in experiment 3 are provided in Figures 7B,C. In addition, LC–MS chromatograms for these earthworms are provided in Supplementary Figure S13 and Supplementary Table S1 with RPAs per sample in Supplementary Figure S13.

**FIGURE 7:**
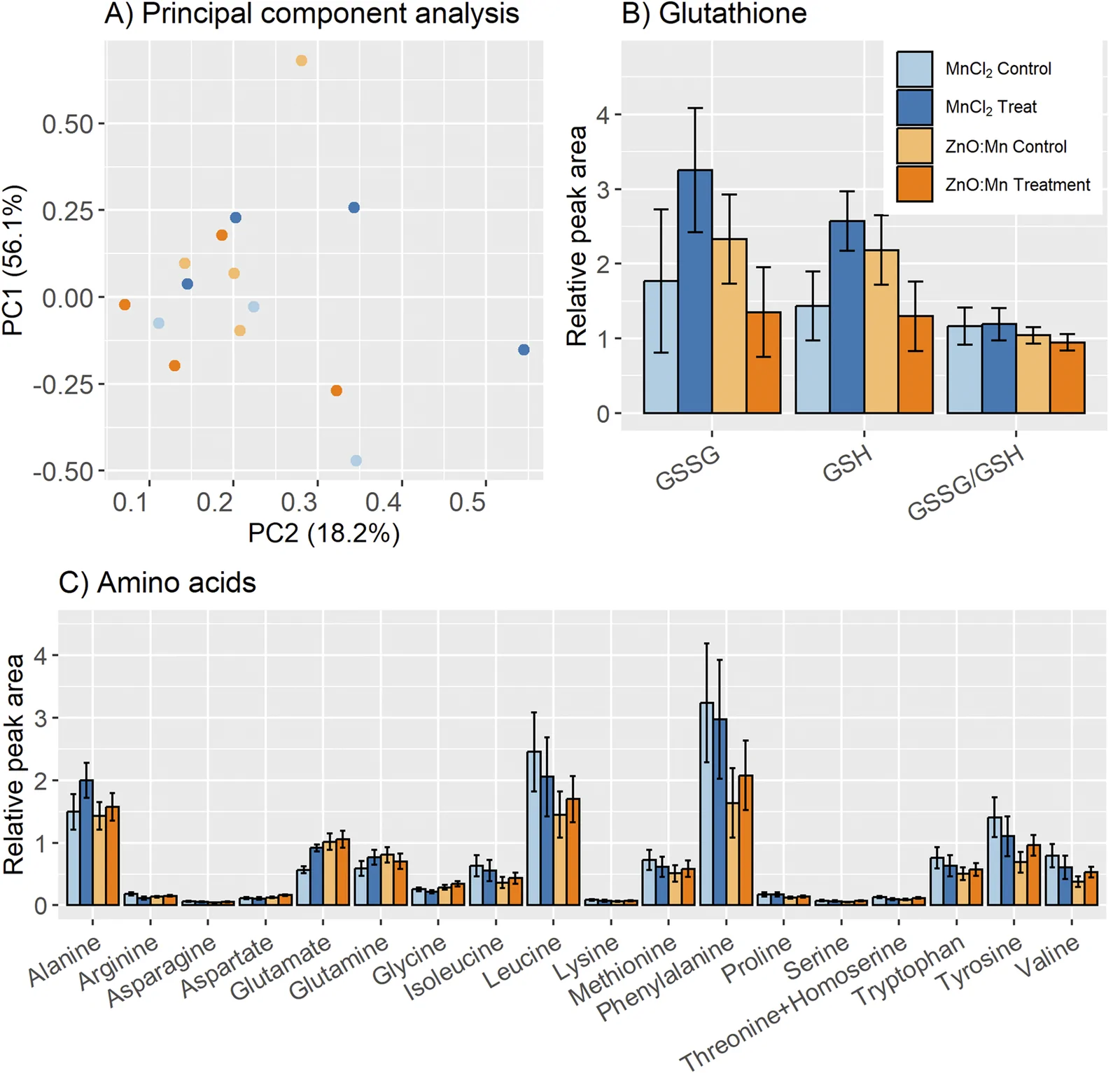
**(A)** Principal component analysis of metabolome in earthworms exposed to MnCl_2_ (blue) and ZnO:Mn (orange) with corresponding control treatments for 14 days in experiment 3; **(B)** relative peak areas (mean ± standard error) for oxidized glutathione (GSSG), reduced glutathione (GSH), the GSSG/GSH ratio, and **(C)** various amino acids.

Glutathione is a tripeptide consisting of cysteine, glycine, and glutamate, and it is a commonly used marker of oxidative stress. GSSG and GSH contribute to a normal redox balance that helps cells survive under stress conditions. Disturbed GSH has been observed in the digestive system of *Pheretima guillemi* earthworms exposed to silver nanoparticles (Li et al., 2022). Despite the biological variability observed between samples of earthworms exposed to ZnO:Mn for 14 days in experiment 3, the mean GSSG/GSH ratios per group presented no significant differences (Figure 7B), with RPAs of 1.05 ± 0.25 for the control and 0.95 ± 0.22 for the ZnO:Mn-treated earthworms indicating no oxidative stress response. The MnCl_2_ control and treatment earthworm samples exhibited more variation in its GSSG and GSH RPAs compared to the ZnO:Mn control and treatment samples, but the GSSG/GSH ratio was similar, and no significant difference between the MnCl_2_-treated and control earthworms was observed.

Glutamate showed a significant increase for MnCl_2_- vs. control-treated earthworms (Figure 7C; P = 0.002), which aligns with previously observed increased glutamate in *E. fetida* earthworms exposed to nanomaterials (Whitfield Åslund et al., 2012). However, glutamate was found decreased in annelid lugworms exposed to ZnO nanoparticles for 14 days (Bruhns et al., 2023). Aspartate tended to be increased for ZnO:Mn- vs. control-treated earthworms (*P* = 0.09), which was also observed to be increased in earthworms exposed to silver nanoparticles in sludge-amended soil (Li et al., 2022). Oxidative and toxic stress responses and increased energy expenditure in earthworms may upregulate metabolic conversions such as aminotransferase of alanine and phenylalanine, which are used for energy generation through the tricarboxylic acid cycle (TCA; Dani et al., 2019). Nonetheless, the present study did not confirm changes in alanine and phenylalanine, as only numerical increases were observed in response to MnCl_2_ exposure for 14 days (Figure 7; Supplementary Data File S4). Leucine was not significantly affected either, despite the well-established role of this amino acid in protein turnover and cellular energy balance under stress conditions (Wang et al., 2018). Overall, the identified amino acids other than glutamate and aspartate were not significantly affected by MnCl_2_ and ZnO:Mn treatments (*P* > 0.10). Further details including normalized metabolome data are available in File S4, and statistical comparisons (ANOVA) across groups such as *P*-values and log_2_ fold-change (log_2_FC) are available in File S5.

Observed early differential gene expression changes after exposure of earthworms to ZnO for 4 days or to ZnO:Mn for 2 days reflected acute responses, while sustained changes in transcriptome or metabolome indicating delayed or adaptive effects were minor after 14 days of exposure to ZnO:Mn and MnCl_2_. In addition, the limited transcriptomic and metabolomic responses that were observed after 14 days of exposure to ZnO:Mn and MnCl_2_ could not be associated to each other, which does not indicate substantial ecotoxicological response in common pathways or gene–metabolite matches. Performing a metabolomic analysis of earthworm samples at earlier time-points than day 14 might have revealed metabolic disturbances aligning closely with transcriptomic shifts, capturing a more dynamic phase of the organism’s response. The coherence of transcriptomic and metabolomic profiling, revealing only minor disturbances without common pathways or gene–metabolite matches in the endpoint observation after 14 days of exposure to ZnO and ZnO:Mn nanomaterials and MnCl_2_, does not indicate substantial ecotoxicity in earthworms. However, the present study did not assess if these minor disturbances remained beyond 14 days of exposure. Longer exposure periods could determine if biochemical responses completely return to baseline levels or a minor adaptive metabolic shift persists.

## 4 Conclusion

Exposing earthworms to ZnO and ZnO:Mn nanomaterials at an inclusion rate of 850 mg/kg soil caused mainly transient transcriptomic responses, with pronounced differential gene expression at day 4 for ZnO and at day 2 for ZnO:Mn. Various GO terms obtained through enrichment analysis of genes that were differentially expressed after exposure to ZnO for 4 days and ZnO:Mn for 2 days could be associated with muscle biology. Exposing mixed-species earthworms to ZnO:Mn nanomaterial and MnCl_2_ indicated that differential gene expression had largely subsided by day 14. These end-point transcriptomic measurements showed moderate differential gene expression with few genes per enriched GO term and no clear link to the detected metabolic disturbances of increased glutamate in response to 14 days of MnCl_2_ exposure and a tendency for increased aspartate after 14 days of ZnO:Mn exposure. The metabolomic profiles provided no evidence of oxidative stress, and the limited overlap between transcriptomic changes and metabolite responses indicates that these effects reflect short-lived physiological adjustments rather than persistent disruption of key biological pathways. Overall, our integrated transcriptomic and metabolomic data indicate that exposure to ZnO and ZnO:Mn nanomaterials under the tested conditions showed no evidence for sustained and substantial ecotoxic effects in earthworms.

## Data Availability

The pipeline for bioinformatics analysis is available through https://git.wur.nl/ssb/publications/gene-expression-profile-dynamics-and-metabolome-of-earthworms-exposed-to-nanomaterials. Datasets presented in this study can be found in the ENA online repository: https://www.ebi.ac.uk/ena/browser/view/PRJEB93781.

## References

[B1] Aulakh M. K. Singh H. Vashisht A. Kang T. S. Sharma R. (2022). Improved photoresponse of sunlight-driven Mn^n+^-ZnO (*n* = 2, 4, 7) nanostructures: a study of oxidative degradation of methylene blue. Top. Catal. 65, 1951–1962. 10.1007/s11244-022-01703-5

[B2] Baccaro M. Harrison S. van den Berg H. Sloot L. Hermans D. Cornelis G. (2019). Bioturbation of Ag_2_S-NPs in soil columns by earthworms. Environ. Pollut. 252, 155–162. 10.1016/j.envpol.2019.05.106 31146230

[B3] Baccaro M. Montaño M. D. Cui X. Mackevica A. Lynch I. von Der Kammer F. (2022). Influence of dissolution on the uptake of bimetallic nanoparticles Au@Ag-NPs in soil organism *Eisenia fetida* . Chemosphere 302, 134909. 10.1016/j.chemosphere.2022.134909 35551940

[B4] Bacchetta R. Maran B. Marelli M. Santo N. Tremolada P. (2016). Role of soluble zinc in ZnO nanoparticle cytotoxicity in *daphnia magna*: a morphological approach. Env. Res. 148, 376–385. 10.1016/j.envres.2016.04.028 27131075

[B5] Bhambri A. Dhaunta N. Patel S. S. Hardikar M. Bhatt A. Srikakulam N. (2018). Large scale changes in the transcriptome of *Eisenia fetida* during regeneration. PLoS ONE 13, e0204234. 10.1371/journal.pone.0204234 30260966 PMC6160089

[B6] Browne W. R. McGarvey J. J. (2007). The raman effect and its application to electronic spectroscopies in metal-centered species: techniques and investigations in ground and excited states. Coord. Chem. Rev. 251, 454–473. 10.1016/j.ccr.2006.04.019

[B7] Bruhns T. Timm S. Sokolova I. M. (2023). Metabolomics-based assessment of nanoparticles (*n*ZnO) toxicity in an infaunal marine annelid, the lugworm *arenicola marina* (annelida: sedentaria). Sci. Tot. Environ. 858, 160039. 10.1016/j.scitotenv.2022.160039 36356734

[B8] Cai J. Bennie J. Gaston K. J. (2025). Altered surface behaviour in earthworms (*Lumbricus terrestris*) under artificial light at night. Oecologia 207, 114. 10.1007/s00442-025-05750-z 40576833 PMC12205020

[B9] Carreira-Barral I. Díez-Hernández J. Igos E. Saidani M. Ding T. da Silva T. R. (2025). Driving sustainability at early-stage innovation in production of zinc oxide nanoparticles. Sust. Prod. Consum. 55, 353–372. 10.1016/j.spc.2025.03.003

[B10] Chandekar K. V. Shkir M. Yadav S. P. P. K. B. AlFaify S. (2022). Facile synthesis of Mn-doped ZnO nanoparticles by flash combustion route and their characterizations for optoelectronic applications. J. Mat. Sci. Mat. Electron 33, 3849–3869. 10.1007/s10854-021-07576-w

[B11] Dani V. Lankadurai B. Nagato E. Simpson A. Simpson M. (2019). Comparison of metabolomic responses of earthworms to sub-lethal imidacloprid exposure in contact and soil tests. Environ. Sci. Pollut. Res. 26, 18846–18855. 10.1007/s11356-019-05302-y 31065987

[B12] Filipiak Z. M. Bednarska A. J. (2021). Different effects of Zn nanoparticles and ions on growth and cellular respiration in the earthworm *Eisenia andrei* after long-term exposure. Ecotoxicol 30, 459–469. 10.1007/s10646-021-02360-2 33616802 PMC7987695

[B13] Gomes S. I. de Boer T. E. van Gestel C. A. van Straalen N. M. Soares A. M. Roelofs D. (2022). Molecular mechanisms of zinc toxicity in the potworm *enchytraeus crypticus*, analysed by high-throughput gene expression profiling. Sci. Tot. Environ. 825, 153975. 10.1016/j.scitotenv.2022.153975 35183636

[B14] Hasan M. Liu Q. Kanwal A. Tariq T. Mustafa G. Batool S. (2024). A comparative study on green synthesis and characterization of Mn doped ZnO nanocomposite for antibacterial and photocatalytic applications. Sci. Rep. 14, 7528. 10.1038/s41598-024-58393-0 38553550 PMC10980724

[B16] Huang J. Xu Q. Sun Z. J. Tang G. L. Su Z. Y. (2007). Identifying earthworms through DNA barcodes. Pedobiologia 51, 301–309. 10.1016/j.pedobi.2007.05.003

[B17] Jaskulak M. Rorat A. Kurianska-Piatek L. Hofman S. Bigaj J. Vandenbulcke F. (2021). Species-specific Cd-detoxification mechanisms in lumbricid earthworms *Eisenia andrei, Eisenia fetida* and their hybrids. Ecotoxicol. Environ. Saf. 208, 111425. 10.1016/j.ecoenv.2020.111425 33068978

[B18] Kanehisa M. Sato Y. Kawashima M. Furumichi M. Tanabe M. (2016). KEGG as a reference resource for gene and protein annotation. Nucleic Acids Res. 44, D457–D462. 10.1093/nar/gkv1070 26476454 PMC4702792

[B19] Li M. Ruan L. Y. Dang F. Lu H. L. Zhou D. M. Yin B. (2022). Metabolic response of earthworms (Pheretima guillemi) to silver nanoparticles in sludge-amended soil. Environ. Pollut. 300, 118954. 10.1016/j.envpol.2022.118954 35122920

[B20] Love M. I. Huber W. Anders S. (2014). Moderated estimation of fold change and dispersion for RNA-seq data with DESeq2. Genome Biol. 15, 550. 10.1186/s13059-014-0550-8 25516281 PMC4302049

[B21] Lu P. J. Huang S. C. Chen Y. P. Chiueh L. C. Shih D. Y. C. (2015). Analysis of titanium dioxide and zinc oxide nanoparticles in cosmetics. J. Food Drug Anal. 23, 587–594. 10.1016/j.jfda.2015.02.009 28911719 PMC9351801

[B22] Novo M. Lahive E. Díez-Ortiz M. Matzke M. Morgan A. J. Spurgeon D. J. (2015). Different routes, same pathways: molecular mechanisms under silver ion and nanoparticle exposures in the soil sentinel *Eisenia fetida* . Environ. Pollut. 205, 385–393. 10.1016/j.envpol.2015.07.010 26204059

[B23] Novo M. Lahive E. Ortiz M. D. Spurgeon D. J. Kille P. (2020). Toxicogenomics in a soil sentinel exposure to Zn nanoparticles and ions reveals the comparative role of toxicokinetic and toxicodynamic mechanisms. Environ. Sci. Nano 7, 1464–1480. 10.1039/C9EN01124B

[B24] Özgür Ü. Hofstetter D. Morkoc H. (2010). ZnO devices and applications: a review of current status and future prospects. Proc. IEEE 98, 1255–1268. 10.1109/JPROC.2010.2044550

[B25] Papadiamantis A. G. Mavrogiorgis A. Papatzelos S. Mintis D. Melagraki G. Lynch I. (2024). A systematic review on the state-of-the-art and research gaps regarding inorganic and carbon-based multicomponent and high-aspect ratio nanomaterials. Comput. Struct. Biotechnol. J. 25, 211–229. 10.1016/j.csbj.2024.10.020 39526292 PMC11550189

[B26] Persaud I. Raghavendra A. J. Paruthi A. Alsaleh N. B. Minarchick V. C. Roede J. R. (2020). Defect-induced electronic states amplify the cellular toxicity of ZnO nanoparticles. Nanotoxicol 14, 145–161. 10.1080/17435390.2019.1668067 31553248 PMC7036006

[B27] Plytycz B. Bigaj J. Rysiewska A. Osikowski A. Hofman S. Podolak A. (2020). Impairment of reproductive capabilities in three subsequent generations of asymmetric hybrids between *Eisenia andrei and E. fetida* from French, Hungarian and Polish laboratory colonies. PLOS ONE 15, e0235789. 10.1371/journal.pone.0235789 32645117 PMC7347191

[B28] Plytycz B. Kruk J. Panz T. SuÅ‚ek M. Hofman S. (2024). Tracking introgression of quinazoline M-Fluorophores from *Eisenia andrei to E. fetida* via interspecific hybrids of these hermaphroditic lumbricid earthworms. J. Biotechnol. Biomed. 7, 360–368. 10.26502/jbb.2642-91280159

[B29] Prashanth G. K. Dileep M. S. Gadewar M. Ghosh M. K. Rao S. Giresha A. S. (2024). Zinc oxide nanostructures: illuminating the potential in biomedical applications: a brief overview. BioNanoSci 14, 1876–1896. 10.1007/s12668-024-01366-4

[B30] Römbke J. Aira M. Backeljau T. Breugelmans K. Domínguez J. Funke E. (2016). DNA barcoding of earthworms (*eisenia fetida/andrei* complex) from 28 ecotoxicological test laboratories. Appl. Soil Ecol. 104, 3–11. 10.1016/j.apsoil.2015.02.010

[B31] Safaei-Ghomi J. Ghasemzadeh M. A. (2017). Zinc oxide nanoparticle promoted highly efficient one pot three-component synthesis of 2, 3-disubstituted benzofurans. Arab. J. Chem. 10, S1774–S1780. 10.1016/j.arabjc.2013.06.030

[B32] Samarasinghe S. V. A. C. Krishnan K. Aitken R. J. Naidu R. Megharaj M. (2023). Chronic effects of TiO_2_ and ZnO nanoparticles to earthworm *Eisenia fetida* . Environ. Chem. Ecotoxicol. 5, 129–134. 10.1016/j.enceco.2023.04.001

[B33] Schurch N. J. Schofield P. Gierliński M. Cole C. Sherstnev A. Singh V. (2016). How many biological replicates are needed in an RNA-seq experiment and which differential expression tool should you use? RNA 22, 839–851. 10.1261/rna.053959.115 27022035 PMC4878611

[B34] Serpone N. Dondi D. Albini A. (2007). Inorganic and organic UV filters: their role and efficacy in sunscreens and suncare products. Inorganica Chim. Acta 360, 794–802. 10.1016/j.ica.2005.12.057

[B35] Shao Y. Wang X. B. Zhang J. J. Li M. L. Wu S. S. Ma X. Y. (2020). Genome and single-cell RNA-sequencing of the earthworm *Eisenia andrei* identifies cellular mechanisms underlying regeneration. Nat. Commun. 11, 2656. 10.1038/s41467-020-16454-8 32461609 PMC7253469

[B36] Sheppard P. S. (1988). “Specific differences in cocoon and hatchling production in *Eisenia fetida* and *E. andrei* ,” in Earthworms in Waste and Environmental Management. Editors Edwards C. A. Neuhauser E. F. (The Hague: SPB), 83–92.

[B37] Singh K. Malla M. A. Kumar A. Yadav S. (2024). Biological monitoring of soil pollution caused by two different zinc species using earthworms. Environ. Sci. Pollut. Res. 31, 57789–57803. 10.1007/s11356-024-34900-8 39292303

[B46] The Gene Ontology Consortium (2019). The gene ontology resource: 20 years and still GOing strong. Nucleic Acids Res. 47, D330–D338. 10.1093/nar/gky1055 30395331 PMC6323945

[B38] Tsyusko O. V. Hardas S. S. Shoults-Wilson W. A. Starnes C. P. Joice G. Butterfield D. A. (2012). Short-term molecular-level effects of silver nanoparticle exposure on the earthworm, *Eisenia fetida* . Environ. Pollut. 171, 249–255. 10.1016/j.envpol.2012.08.003 22960366

[B39] Van Lingen H. J. Saccenti E. Suarez-Diez M. Baccaro M. van den Brink N. W. (2025). Predicting uptake and elimination kinetics of chemicals in invertebrates: a technical note on residual variance modeling. Comput. Toxicol. 33, 100337. 10.1016/j.comtox.2024.100337

[B40] Wang H. Wang J. Zhang Z. (2018). Leucine exerts lifespan extension and improvement in three types of stress resistance (thermotolerance, anti-oxidation and anti-UV irradiation) in *C. elegans* . J. Food Nutr. Res. 6, 665–673. 10.12691/jfnr-6-10-8

[B41] Whitfield Åslund M. Simpson M. J. Simpson A. J. Zeeb B. A. Rutter A. (2012). Earthworm metabolomic responses after exposure to aged PCB contaminated soils. Ecotoxicol 21, 1947–1956. 10.1007/s10646-012-0928-x 22623111

[B42] Wu T. Hu E. Xu S. Chen M. Guo P. Dai Z. (2021). clusterProfiler 4.0: a universal enrichment tool for interpreting omics data. Innovation 2, 100141. 10.1016/j.xinn.2021.100141 34557778 PMC8454663

[B43] Yu W. Zhang Y. Sang W. (2022). Integration of transcriptomic and metabolomic reveals metabolic pathway alteration in earthworms (*Eisenia fetida*) under copper exposure. Comp. Biochem. Physiol. Part C Toxicol. and Pharmacol. 260, 109400. 10.1016/j.cbpc.2022.109400 35753647

[B44] Zhang F. Wang Z. Peijnenburg W. J. Vijver M. G. (2022). Review and prospects on the ecotoxicity of mixtures of nanoparticles and hybrid nanomaterials. Environ. Sci. Technol. 56, 15238–15250. 10.1021/acs.est.2c03333 36196869 PMC9671040

[B45] Zhu Y. Wu X. Liu Y. Zhang J. Lin D. (2020). Integration of transcriptomics and metabolomics reveals the responses of earthworms to the long-term exposure of TiO_2_ nanoparticles in soil. Sci. Tot. Environ. 719, 137492. 10.1016/j.scitotenv.2020.137492 32120103

