# Supplementary material for: Integrated transcriptomic and metabolomic ecotoxicity assessment of earthworms exposed to ZnO and Mn-doped ZnO nanomaterials
Source: Front Toxicol. 2026 Sep 23;8:1927574. doi: 10.3389/ftox.2026.1927574 (PMC13641736; doi:10.3389/ftox.2026.1927574)
Supplement: Supplementary file 1 [file Supplementaryfile1.docx]

Integrated Transcriptomic and Metabolomic Ecotoxicity Assessment of Earthworms Exposed to ZnO and Mn-Doped ZnO Nanomaterials - Supplementary Material

Henk J. van Lingen, ^a^ Paraskevi Zagana, ^b^ Changlin Ke, ^a^ Edoardo Saccenti, ^a^ Maria Klapa, ^b^ Zoi G. Lada,^d^ Marta Baccaro, ^c^ Nico W. van den Brink, ^c^ and Maria Suarez-Diez ^a,*^

a) Laboratory of Systems & Synthetic Biology, Wageningen University & Research, Wageningen, The Netherlands
b) Metabolic Engineering and Systems Biology Laboratory, Institute of Chemical Engineering Sciences, Foundation for Research and Technology-Hellas (FORTH/ICE-HT), Patras, Greece
c) Division of Toxicology, Wageningen University & Research, Wageningen, The Netherlands
d) Department of Chemistry, Laboratory of Applied Molecular Spectroscopy, Institute of Chemical Engineering Sciences (ICEHT), Foundation for Research and Technology-Hellas (FORTH/ICE-HT), University of Patras, Patras, Greece

# 1 Supplementary Materials & Methods

## 1.1 Experimental details of ZnO and ZnO:Mn nanomaterials characterization

Raman spectra were recorded on a T-64000 (Jobin Yvon-Horiba) micro-Raman system equipped with a 2D-CCD Symphony II detector. The excitation wavelength (514.5 nm) was provided by a DPSS laser (Cobolt Fandango TMISO laser). Dispersion and detection of the Raman photons were done by a 600 grooves/mm grating and a Spectraview-2DTM liquid N_2_-cooled CCD detector, respectively. The laser was focused on the samples by a 50x microscope objective with a power of 1 mW on sample. The resolution was kept constant in all experiments (~4 cm^-1^). Raman peak interpretation of Mn-doped ZnO oxides, specifically those at 530, 570, and 675 cm⁻¹ is described by Hu et al. (2011). XRD spectra were performed for the structural characterization of ZnO and ZnO:Mn samples by using a Bruker D8 Advance diffractometer equipped by a Cu lamp (λCuKa = 1.54046 Å) at a scanning rate 0.5 sec/step over a range 2–80^o^ (2θ). SEM images were obtained using a Zeiss SUPRA 35VP-FEG instrument, operating at 5–20 keV. The system is equipped with EDX (Bruker GmbH, Quanta 200) and BSE (K E Developments), which enabled elemental analyses.

## 1.2 Zinc and manganese content determination in soil samples

All 32 and 18 oven dried soil samples from emptied jars in experiments 1 and 2, respectively, were ground at 500 µm and then dissolved using aqua regia (3:1 ratio of HCl:HNO_3_). Then, after digestion and atomization, the concentrations of the corresponding elements were determined using inductively coupled plasma optical emission spectrometry (Thermo iCAP-6500 DV, Thermo Fisher Scientific; ICP-OES). The ICP-OES measurements were conducted in accordance with the guidelines in NPR-6425 and NEN-6966. Internal standards elements of the ICP-OES analysis included Be, Sc and Y. Grinding, further processing and metal content determination were performed by the soil chemistry laboratory (CBLB) at Wageningen University. Zn and Mn extraction from the 16 soil samples from experiment 3 was performed using Teflon vessels and 7 mL of reverse aqua regia (3:1 ratio of HCl:HNO_3_; Merck, Darmstadt) on hot plates in an open system. After diluting properly, the digested samples were analyzed by inductively coupled plasma-mass spectrometry (ICP-MS Nexion 350D, Perkin-Elmer Inc., Waltham, MA). The calibration curve was prepared from solutions of Zn^2+^ and Mn^2+^ (standard stock solution 1000 mg/L of ionic metal ion, Merck, Darmstadt) in a matching matrix.

## 1.3 RNA extraction and sequencing

Ground earthworm samples from experiments 1 and 2 were sent to BMKGene (Münster, Germany) for RNA extraction and sequencing. A TRIzol kit was used to complete the extraction of RNA, after which the concentration of extracted nucleic acid was detected using a Nanodrop2000 spectrophotometer (Thermo) and the integrity with an Agient2100 Bioanalyzer LabChip GX (Perkin Elmer). Libraries from poly(A) enriched RNA were prepared along purification, fragmentation, reverse transcription to cDNA, ligation and amplification using a Hieff NGS Ultima Dual-mode mRNA Library Prep Kit for Illumina (Yeasen; model:13533ES96) OR VAHTS Universal V8 RNA-seq library preparation kit for Illumina – NR605. Prepared libraries were inspected by Qsep-400 and then sequenced on an Illumina Novaseq X platform (Illumina, San Diego, CA) and reads were generated using paired-end 2×150 base pairs.

Ground samples of two paired earthworms from experiment 3 were sent to GENEWIZ Germany GmbH (Leipzig, Germany) for RNA extraction and sequencing using the same requirements as for the orders of experiments 1 and 2 sent to BMKgene. Qiagen RNeasy Mini kit (Qiagen, Hilden, Germany) was used to complete the extraction of RNA. The concentration of extracted RNA was detected using a Qubit 4.0 Fluorometer (Life Technologies, Carlsbad, CA, USA) and the integrity was checked with RNA Kit on Agilent 5300 Fragment Analyzer (Agilent Technologies, Palo Alto, CA, USA). Libraries from poly(A) enriched RNA were prepared along purification, fragmentation, reverse transcription to cDNA, ligation and amplification using the NEBNext Ultra II RNA Library Prep Kit for Illumina (NEB, Ipswich, MA, USA). Sequencing libraries were validated using NGS Kit on the Agilent 5300 Fragment Analyzer (Agilent Technologies, Palo Alto, CA, USA), and quantified using a Qubit 4.0 Fluorometer (Invitrogen, Carlsbad, CA). The sequencing libraries were multiplexed and loaded on the flow cell on the Illumina NovaSeq 6000 instrument using paired-end 2×150 base pairs.

## 1.4 Raw reads quality control for all samples

The raw Illumina paired-end reads (The fasta files are available through the European Nucleotide Archive (ENA) at EMBL-EBI under accession number PRJEB93781; https://www.ebi.ac.uk/ena/browser/view/PRJEB93781) were processed using the workflow described at: <https://workflowhub.eu/workflows/336?version=1>, which was developed with the Common Workflow Language (CWL) v1.2. The workflow was used with default settings plus rRNA removal based on ribo-Kmers from SILVA (Bushnell, 2014; ribo-Kmers were downloaded from: <https://portal.nersc.gov/dna/microbial/assembly/bushnell/>) to perform quality control, trimming, filtering, and classification to ensure high-quality input for downstream analyses. Optional steps in the workflow were omitted.

For experiments 1 and 2, raw fastq reads underwent pre-quality control (QC) at sequencing facility using in-house Perl scripts and the fastp tool at BMKgene. The pre-QC process involved filtering steps that removed adapter sequences, poly-N stretches, and low-quality reads (phred score < 10 for 10 bp, as per fastp parameters: -Q -y -g -Y 10 -l 100 -b 150 -B 150). A minimum Q30 threshold of 85% was enforced for high-quality reads. The resulting "clean" reads (_good.fastq files) were then further processed through our QC pipeline. For all experiments (“clean reads” of experiment 1 and 2 and raw reads of experiment 3), docker containers were used throughout our custom QC pipeleine to ensure reproducibility. Initial and after-filtering quality check was performed using FastQC v0.11.9 (Andrews, 2010). Reads were then trimmed with Fastp v0.20.1 (Chen et al., 2018), which corrected mismatched base pairs in overlapped regions of paired-end reads to improve overall read accuracy. Additionally, reads shorter than 50 bp were removed. Low-quality bases were filtered out by requiring minimum Phred score Q20 and reads with more than 20% low-quality bases were discarded. BBduk v38.87 (Bushnell, 2014) was used to remove non-target sequences such as ribosomal RNA (rRNA) and phiX. To check for potential contaminants, taxonomic classification was performed using Kraken2 with full PlusPFP-16 database, followed by refinement of abundance estimates using Bracken. The results from these quality controls and all subsequent analyses were aggregated using multiQC v1.9 (Ewels et al., 2016.

## 1.5 Read alignment and quantification for all samples

Assembled and annotated genomes of *Eisenia andrei* and *Eisenia fetida*, which are morphologically similar earthworms, were retrieved from National Genomics Data Center (Genome Warehouse, GWH; http://bigd.big.ac.cn/gwh/) with accession number GWHACBE00000000 (Shao et al., 2020) and from NCBI with accession number GCA_003999395.1 (Bhambri et al., 2018). Cleaned reads were mapped to the genomes of these species. Samples with mapping rates above 40% were selected for reference-based transcript quantification using FeatureCounts v2.0.1 (Liao et al., 2014).

## 1.6 Reference-free assembly and read counting

Samples with mapping rates to references < 40% were found in experiment 3. For those samples, *de novo* transcriptome assembly was performed using Trinity 2.15.1 (Grabherr et al., 2011). Parameters were set to handle strand-specific paired-end data, ensuring accurate transcript orientation. Low-expressed (minimum TPM < 3) and short transcripts (length < 250 bp) were filtered to enhance assembly quality and reduce redundancy. Post-assembly clustering was applied using CD-HIT-EST 4.8.1 (identify threshold = 95) to collapse similar transcripts while preserving biologically relevant isoforms (Li & Godzik, 2006). Coding regions in the output assembly were predicted by TransDecoder 5.5.0 on Galaxy (<https://github.com/TransDecoder/TransDecoder>). The cleaned reads were then aligned back to the filtered assembly and Trinity-grouped genes and transcripts abundance estimation were conducted with Salmon v0.14.1 (Patro et al., 2017). The assembled transcripts were annotated using Trinotate 4.0.2 (Griffith et al., 2015) to assign putative functions. The annotation pipeline included diamond 2.1.10 BLAST (Buchfink et al., 2015) searches against the SwissProt database (both BLASTX and BLASTP) and Pfam domain identification for protein family assignments. Additionally, EggnogMapper v2.1.12 (Cantalapiedra et al., 2021) was used for orthology-based annotations to assign gene ontology (GO) enrichment terms and KEGG pathways.

## 1.7 Regression-based gene expression downstream analysis

Furthermore, a regression-based analysis was performed that used a generalized linear model specifically suited for the estimation of parameters of serial data using time as an independent quantitative (rather than qualitative as used in the differential expression analysis above) variable. The time series data generated for the nanomaterial and control treatments in experiments 1 and 2 were also fitted to a negative-binomial model in this regression-based analysis. The model fitting procedure was performed along a step in which genes were selected, a step that included backward selection of time and treatment variables with non-zero regression coefficients, and a step that only retained Genes of models with R^2^ > 0.7. Following these three steps, retained genes were subdivided into two groups using the hierarchical clustering method. The regression-based analysis and clustering were performing using the maSigPro package version 1.70.0 in R (Conesa et al., 2006; Nueda et al., 2014).

## 1.8 Chemicals and Reagents used for metabolomics analysis

HPLC grade water & HPLC grade methanol; Norleucine 1mg/ml, Theofylline 0.1 mg/mL, and Ribitol 1mg/mL (as internal standards); Vortex mixer (LabTech, LVM -202); Analytical balance; Automatic pipettes; Water bath; Cooling centrifuge (Thermo Fisher Scientific, Waltham, MA, USA); Vacuum concentrator (Thermo Fisher Scientific, Waltham, MA, USA); FREEDOM Evo, by Tecan (Liquid Handler); Surveyor LC pump Liquid Chromatograph (Thermo Finnigan); LCQ Advantage ion trap mass spectrometer (Thermo Finnigan); Luna C18 Phenomenex column; Nitrogen 99,99 carrier gas; Helium 99.99% carrier gas (Air Liquide Hellas, Athens, Greece).

## 1.9 Metabolomics metabolite extraction

Metabolite extraction followed a modified tissue extraction protocol using HPLC-grade methanol:water (1:1 v/v) as the solvent. Norleucine and theophylline were used as internal standards in a 1:2 ratio (Papadimitropoulos et al., 2018). 100 mg of homogenized tissue was transferred to a new frozen falcon tube, and internal standards were added, followed by cold methanol. The mixture was vortexed gently, incubated at 70 °C for 15–20 minutes, and then diluted with an equal volume of water. Samples were centrifuged at 10,000 rpm for 10 minutes (Cooling centrifuge; Thermo Fisher Scientific, Waltham, MA, USA) until clear supernatants were obtained. After three centrifugation rounds, supernatants were dried under vacuum in a SpeedVac (Thermo Fisher Scientific) and stored at 4 °C for further analysis. Quality Control (QC) samples were prepared combining equal supernatant volumes from all biological samples before drying.

## 1.10 LC-MS Analysis and Data Acquisition

For LC-MS analysis, dried metabolite extracts were re-dissolved in HPLC-grade methanol:water (1:9 v/v) and analyzed using a Surveyor LC pump (Thermo Finnigan) using Nitrogen:Helium 99.99% carrier gas (Air Liquide Hellas, Athens, Greece) coupled with an LCQ Advantage ion trap mass spectrometer (Thermo Finnigan). Metabolites were separated on a Luna C18 Phenomenex column, with polar compounds eluting first. The system operated in positive electrospray ionization mode (ESI, +4.5 kV) and scanned m/z values between 50 and 800. Mobile phases included 0.1% formic acid in i) ultrapure water (Phase A) and ii) methanol (Phase B) at a flow rate of 0.5 mL/min, with the column temperature set at 350 °C. Samples were injected in triplicate, and a quality control (QC) sample was profiled daily or every other day to monitor instrument conditions and normalize data. This ensured consistent operational performance across all sequential runs of the biological samples.

## 1.11 Metabolite Identification

Metabolite peak identification was performed based on the commercial NIST and on the in- house MESBL peak library. The latter is populated by peaks identified based on analysis of standard compounds and peaks from previous experiments in the laboratory compared with NIST and other publicly available databases. The analysis of the profiles was carried out using the Thermo Scientific™ Xcalibur™ Software. The total peak area of each metabolite was estimated from the summation of all peaks corresponding to this metabolite (ion adducts and fragments).

# 2 Supplementary Results

## 2.1 Gene expression time-dependent analysis

A regression-based approach was used to identify differences in the temporal patterns of gene expression across treatments for experiments 1 and 2. A regression-based generalized linear model was established that evaluated time as a quantitative variable in contrast to the differential expression analysis in which time was considered a qualitative variable. 73 out of 28,365 genes were identified with significant differences in their temporal patters for experiment 1. Clustering resulted in 41 genes that showed increased expression on days 3 and 7 for ZnO treated earthworms relative to the control treated earthworms (Fig. S11, cluster 1A). A second cluster of 32 genes indicated an increase in gene expression in ZnO treated relative to control treated earthworms on days 3 and 7 from the start of the exposure (Fig. S11, cluster 1B). Furthermore, more than 1000 out of 28,241 genes were identified as showing differences in their gene expression temporal dynamics upon exposure to control or ZnO:Mn treated soil in experiment 2. Clustering resulted in a first cluster consisting of 501 genes (Fig. S11 cluster 2A) that contained genes showing increased expression for the ZnO:Mn treated earthworms on day 2 from the start of the exposure, whereas similar gene expression was observed on days 4 and 7. A second cluster of 532 genes indicated downregulated expression in earthworms exposed to ZnO:Mn relative to earthworms that had received the control treatment for 2 days, but this downregulation decreased along days 4 and 7 (Fig. S11, cluster 2B). This difference observed in gene expression for day 2 using regression aligns with the strong transcriptional response indicated by the differential expression analysis on day 2 samples.

# 3 Supplementary Figures and Tables

## 3.1 Supplementary Figures

|  |  |
| --- | --- |
| **Supplementary Figure 1.** Raman spectra (a) and X-ray diffraction (b) of ZnO and ZnO:Mn samples. | |

|  |  |
| --- | --- |
|  | |
| Supplementary Figure 2. Representative SEM images of samples. | |

|  |  |
| --- | --- |
| Supplementary Figure 3. SEM-EDX images for ZnO:Mn sample. | |

**Supplementary Figure 4**. Zinc and manganese contents (mg/kg) in samples of ZnO treated and control soil samples in experiment 1.

**Supplementary Figure 5.** Zinc and manganese contents (mg/kg) in samples of ZnO:Mn treated and control soil samples in experiment 2.

**Supplementary Figure 6.** Zinc and manganese contents (mg/kg) in samples of ZnO:Mn treated, control treated and MnCl_2_ treated soil samples in experiment 3.

**Supplementary Figure 7.** Heatmap for correlations of ~29,000 gene transcripts mapped to *E. andrei* for experiment 1.

**Supplementary Figure 8.** Heatmap for correlations of ~29,000 gene transcripts mapped to *E. andrei* for experiment 2.

**Supplementary Figure 9.** Heatmap for correlations of ~73,000 *de novo* assembled gene transcripts for experiment 3.

**Supplementary Figure 10.** Volcano plots that represent differential gene expression of transcripts mapped to the *E. andrei* reference genome for earthworms exposed to ZnO treated soil for 4 days *vs*. earthworms exposed to ZnO treated soil for 2, 3 and 7 days in experiment 1 (a-c).

**Supplementary Figure 11.** Regression-based profiles of median expression for clusters of selected genes obtained from transcripts mapped to the *E. andrei* reference genome from earthworms exposed to ZnO *vs* control soil for 2, 3 and 7 days in experiment 1 (clusters 1A-B), and exposed to ZnO:Mn *vs* control soil for 2, 4 and 7 days in experiment 2 (clusters 2A-B).

**Supplementary Figure 12.** Representative LC-MS chromatograms for the control and treated earthworm samples. Peak heights represent the relative abundance in the sample and are not yet normalized to earthworm weight of each sample or the norleucine internal standard.

**Supplementary Figure 13.** Metabolite levels in earthworms exposed to MnCl₂ and ZnO:Mn nanomaterials for 14 days compared to their respective controls. The plots represent the relative peak areas of metabolites in all samples across the MnCl_2_-control, MnCl₂-treatment, ZnO:Mn-control, and ZnO:Mn-treatment groups (a-d). Each group consisted of four biological samples (pairs of two earthworms).

## 3.2 Supplementary Figures

**Supplementary Table 1.** Identification of amino acids by LC-MS

| **Metabolite** | **MW, g/mol** | **Average Retention Time (min) ± sd** | **Average m/z ± sd** |
| --- | --- | --- | --- |
| L-Lysine* | 146.19 | 2.30 ± 0.02 | 146.9 ± 0.0 |
| L-Arginine* | 174.20 | 2.48 ± 0.05 | 175.2 ± 0.2 |
| L-Glycine* | 75.07 | 2.89 ± 0.02 | 76.07 ± 0.2 |
| L-Alanine* | 89.09 | 2.96 ± 0.02 | 89.9 ± 0.0 |
| L-Asparagine | 132.12 | 2.94 ± 0.02 | 133.0 ± 0.0 |
| L-Serine* | 105.09 | 2.93 ± 0.02 | 105.9 ± 0.0 |
| L-Glutamine | 147.14 | 2.99 ± 0.01 | 147.9 ± 0.0 |
| L-Threonine / Homoserine** | 119.12 | 3.04 ± 0.02 | 120.0 ± 0.1 |
| L-Glutamate* | 147.13 | 3.09 ± 0.01 | 147.9 ± 0.0 |
| L-Aspartate* | 133.10 | 3.19 ± 0.08 | 133.9 ± 0.1 |
| L-Proline* | 115.13 | 3.35 ± 0.02 | 116.0 ± 0.0 |
| L-Valine* | 117.15 | 3.76 ± 0.01 | 117.9 ± 0.0 |
| L-Methionine* | 149.21 | 4.62 ± 0.02 | 149.9 ± 0.1 |
| L-Tyrosine* | 181.19 | 6.04 ± 0.05 | 181.9 ± 0.0 |
| L-Isoleucine* | 131.17 | 6.18 ± 0.04 | 131.8 ± 0.1 |
| L-Leucine* | 131.17 | 6.75 ± 0.04 | 131.9 ± 0.2 |
| L-Phenylalanine* | 165.19 | 13.33 ± 0.08 | 166.0 ± 0.0 |
| L-Tryptophan | 204.22 | 19.90 ± 0.07 | 204.9 ± 0.0 |
|  |  |  |  |
| **Internal Standard** | | | |
| L-Norleucine* | 131.17 | 7.29 ± 0.04 | 131.9 ± 0.0 |

**Supplementary Table 2.** Mean Relative Peak Areas of GSSG and GSH and GSH/GSSG ratio across **MnCl_2_ treatment** group and its **control** group. GSSG and GSH RPA values are the mean values of three technical replicates (% Coefficient of Variation in parenthesis)

| **Group** | **MnCl_2_ control** | | | | **MnCl_2_ treatment** | | | |
| --- | --- | --- | --- | --- | --- | --- | --- | --- |
| **Sample** | **MnCl_2_-Ctrl-1** | **MnCl_2_-Ctrl-2** | **MnCl_2_-Ctrl-3** | **MnCl_2_-Ctrl-4** | **MnCl_2_-Treat-1** | **MnCl_2_-Treat-2** | **MnCl_2_-Treat-3** | **MnCl_2_-Treat-4** |
| GSSG RPA per sample | 2.62 ± 0.41 (16%) | 2.03 ± 0.06 (3%) | 0.65 ± 0.05 (7%) | Not detected | 5.72 ± 0.33 (6%) | 2.17 ± 0.26 (12%) | 3.89 ± 0.37 (10%) | 1.23 ± 0,10 (8%) |
| GSH RPA per sample | 1.59 ± 0.20 (12%) | 1.75 ± 0.13 (7%) | 0.96 ± 0.05 (5%) | Not detected | 3.24 ± 0.33 (10%) | 2.08 ± 0.15 (7%) | 3.50 ± 0.47 (14%) | 1.46 ± 0.20 (14%) |
| GSSG/GSH ratio per sample | 1.68 ± 0.47 (28%) | 1.16 ± 0.08 (7%) | 0.68 ± 0.03 (5%) | x | 1.77 ± 0.10 (6%) | 1.04 ± .07 (7%) | 1.12 ± 0.05 (4%) | 0.86 ± 0.14 (17%) |
| Mean GSSG/GSH ratio per group | 1.18 ± 0.49 (42%) | | | | 1.20 ± 0.37 (31%) | | | |

**Supplementary Table 3.** Mean Relative Peak Areas of GSSG and GSH/GSSG ratio across **ZnO:Mn treatment** group and its **control** group. GSSG and GSH RPA values are the mean values of three technical replicates (% Coefficient of Variation in parenthesis)

| **Group** | **ZnO:Mn control** | | | | **ZnO:Mn treatment** | | | |
| --- | --- | --- | --- | --- | --- | --- | --- | --- |
| **Sample** | **ZnO:Mn-Ctrl-1** | **ZnO:Mn-Ctrl-2** | **ZnO:Mn-Ctrl-3** | **ZnO:Mn-Ctrl-4** | **ZnO:Mn-Treat-1** | **ZnO:Mn-Treat-2** | **ZnO:Mn-Treat-3** | **ZnO:Mn-Treat-4** |
| GSSG RPA per sample | 1.22 ± 0,14 (11%) | 1.96 ± 0.12 (6%) | 1.85 ± 0.09 (5%) | 4.29 ± 0.31 (7%) | 0.28 ± 0.02 (6%) | 0.66 ± 0.07 (11%) | 2.32 ± 0.21 (9%) | 2.14 ± 0.30 (14%) |
| GSH RPA per sample | 1.62 ± 0.13 (8%) | 1.96 ± 0,15 (8%) | 1.49 ± 0.29 (19%) | 3.66 ± 0.36 (10%) | 0.44 ± 0.04 (10%) | 0.72 ± 0.04 (5%) | 2.15 ± 0.34 (16%) | 1.88 ± 0.26 (14%) |
| GSSG/GSH ratio per sample | 0.76 ± 0.03 (4%) | 1.00 ± 0.03 (3%) | 1.28 ± 0.33 (26%) | 1.17 ± 0.04 (3%) | 0.64 ± 0.11 (17%) | 0.93 ± 0.07 (8%) | 1.09 ± 0.08 (7%) | 1.14 ± 0.12 (11%) |
| Mean GSSG/GSH ratio per group | 1.05 ± 0.25 (24%) | | | | 0.95 ± 0.22 (23%) | | | |

# 4 Supplementary References

Andrews, S. (2010). FastQC: A quality control tool for high throughput sequence data. Available online: <http://www.bioinformatics.babraham.ac.uk/projects/fastqc>.

Bhambri, A., Dhaunta, N., Patel, S.S., Hardikar, M., Bhatt, A., Srikakulam, N., Shridhar, S., Vellarikkal, S., Pandey, R., Jayarajan, R. and Verma, A. 2018. Large scale changes in the transcriptome of *Eisenia fetida* during regeneration. *PLoS ONE* 13, e0204234. <https://doi.org/10.1371/journal.pone.0204234>

Buchfink, B., Xie, C., & Huson, D.H. 2015. Fast and sensitive protein alignment using DIAMOND. *Nature methods* 12, 59-60. <https://doi.org/10.1038/nmeth.3176>

Bushnell, B. (2014). BBMap: A fast, accurate, splice-aware aligner. Available online: <https://sourceforge.net/projects/bbmap/>

Cantalapiedra, C.P., Hernández-Plaza, A., Letunic, I., Bork, P., & Huerta-Cepas, J. 2021. eggNOG-mapper v2: functional annotation, orthology assignments, and domain prediction at the metagenomic scale. *Mol. Biol. Evol.* 38, 5825-5829. <https://doi.org/10.1093/molbev/msab293>

Chen, S., Zhou, Y., Chen, Y., & Gu, J. (2018). fastp: an ultra-fast all-in-one FASTQ preprocessor. *Bioinformatics*, 34(17), i884-i890. <https://doi.org/10.1093/bioinformatics/bty560>

Conesa, A., Nueda, M. J., Ferrer, A., & Talón, M. 2006. maSigPro: a method to identify significantly differential expression profiles in time-course microarray experiments. *Bioinformatics* 22, 1096-1102.

Ewels, P., Magnusson, M., Lundin, S., & Käller, M. (2016). MultiQC: summarize analysis results for multiple tools and samples in a single report. *Bioinformatics*, 32(19), 3047-3048. <https://doi.org/10.1093/bioinformatics/btw354>

Grabherr, M.G., Haas, B.J., Yassour, M., Levin, J.Z., Thompson, D.A., Amit, I., Adiconis, X., Fan, L., Raychowdhury, R., & Zeng, Q. 2011. Trinity: reconstructing a full-length transcriptome without a genome from RNA-Seq data. *Nature Biotechnol.* 29, 644. <https://doi.org/10.1038/nbt.1883>

Griffith, M., Walker, J.R., Spies, N.C., Ainscough, B.J., & Griffith, O.L. 2015. Informatics for RNA sequencing: a web resource for analysis on the cloud. *PLoS Comput. Biol.* 11, e1004393. <https://doi.org/10.1371/journal.pcbi.1004393>

Hu, Y.M., Wang, C.Y., Lee, S.S., Han, T.C., Chou, W.Y. and Chen, G.J. (2011). Identification of Mn-related Raman modes in Mn-doped ZnO thin films. *Journal of Raman Spectroscopy*, 42: 434-437. <https://doi.org/10.1002/jrs.2695>

Li, W., & Godzik, A. 2006. Cd-hit: a fast program for clustering and comparing large sets of protein or nucleotide sequences. *Bioinformatics* 22, 1658-1659. <https://doi.org/10.1093/bioinformatics/btl158>

Liao, Y., Smyth, G.K., & Shi, W. 2014. featureCounts: an efficient general purpose program for assigning sequence reads to genomic features. *Bioinformatics* 30, 923-930. <https://doi.org/10.1093/bioinformatics/btt656>

NEN-6966: 2005. Milieu: Analyse van geselecteerde elementen in water, eluaten en destruaten. Atomaire emissiespectrometrie met inductief gekoppeld plasma (ICP-OES).

NPR-6425: 1995. Praktijkrichtlijn: Atomaire-emissiespectrometrie met inductief gekoppeld plasma. Algemene richtlijnen.

Nueda, M.J., Tarazona, S., & Conesa, A. 2014. Next maSigPro: updating maSigPro bioconductor package for RNA-seq time series. *Bioinformatics* 30, 2598-2602. <https://doi.org/10.1093/bioinformatics/btu333>

Özgür, Ü., Hofstetter, D., & Morkoc, H. 2010. ZnO devices and applications: a review of current status and future prospects. *Proc. IEEE*, 98, 1255-1268. <https://doi.org/10.1109/JPROC.2010.2044550>

Papadimitropoulos M.P., Vasilopoulou C.G., Maga-Nteve C., Klapa MI. 2018. Untargeted GC-MS Metabolomics. *Methods in Mol. Biol.* 1738, 133-147. <https://doi.org/10.1007/978-1-4939-7643-0_9>

Patro, R., Duggal, G., Love, M.I., Irizarry, R.A., & Kingsford, C. 2017. Salmon provides fast and bias-aware quantification of transcript expression. *Nature methods* 14, 417-419. <https://doi.org/10.1038/nmeth.4197>

Shao Y., Wang X.B., Zhang J.J., Li M.L., Wu S.S., Ma X.Y., Wang X., Zhao H.F., Li Y., Zhu H.H., Irwin D.M., Wang D.P., Zhang G.J., Ruan J., Wu D.D. 2020. Genome and single-cell RNA-sequencing of the earthworm Eisenia andrei identifies cellular mechanisms underlying regeneration. *Nature Commun.* 11, 2656. <https://doi.org/10.1038/s41467-020-16454-8>
